# Single-Molecule, Super-Resolution, and Functional Analysis of G Protein-Coupled Receptor Behavior Within the T Cell Immunological Synapse

**DOI:** 10.3389/fcell.2020.608484

**Published:** 2021-01-18

**Authors:** James H. Felce, Lucia Parolini, Erdinc Sezgin, Pablo F. Céspedes, Kseniya Korobchevskaya, Mathew Jones, Yanchun Peng, Tao Dong, Marco Fritzsche, Dirk Aarts, John Frater, Michael L. Dustin

**Affiliations:** ^1^Kennedy Institute of Rheumatology, University of Oxford, Oxford, United Kingdom; ^2^Peter Medawar Building for Pathogen Research, Nuffield Department of Medicine, University of Oxford, Oxford, United Kingdom; ^3^Medical Research Council Human Immunology Unit, Medical Research Council Weatherall Institute of Molecular Medicine, University of Oxford, Oxford, United Kingdom; ^4^Science for Life Laboratory, Department of Women's and Children's Health, Karolinska Institutet, Stockholm, Sweden; ^5^Chinese Academy of Medical Sciences Oxford Institute, Nuffield Department of Medicine, University of Oxford, Oxford, United Kingdom; ^6^Rosalind Franklin Institute, Didcot, United Kingdom; ^7^Department of Chemistry, Physical and Theoretical Chemistry Laboratory, University of Oxford, Oxford, United Kingdom; ^8^National Institute of Health Research Biomedical Research Centre, Oxford, United Kingdom

**Keywords:** lymphocyte, synapse, fluorescence, microscopy, receptor, tracking, signaling, screening

## Abstract

A central process in immunity is the activation of T cells through interaction of T cell receptors (TCRs) with agonistic peptide-major histocompatibility complexes (pMHC) on the surface of antigen presenting cells (APCs). TCR-pMHC binding triggers the formation of an extensive contact between the two cells termed the immunological synapse, which acts as a platform for integration of multiple signals determining cellular outcomes, including those from multiple co-stimulatory/inhibitory receptors. Contributors to this include a number of chemokine receptors, notably CXC-chemokine receptor 4 (CXCR4), and other members of the G protein-coupled receptor (GPCR) family. Although best characterized as mediators of ligand-dependent chemotaxis, some chemokine receptors are also recruited to the synapse and contribute to signaling in the absence of ligation. How these and other GPCRs integrate within the dynamic structure of the synapse is unknown, as is how their normally migratory Gαi-coupled signaling is terminated upon recruitment. Here, we report the spatiotemporal organization of several GPCRs, focusing on CXCR4, and the G protein Gαi2 within the synapse of primary human CD4^+^ T cells on supported lipid bilayers, using standard- and super-resolution fluorescence microscopy. We find that CXCR4 undergoes orchestrated phases of reorganization, culminating in recruitment to the TCR-enriched center. This appears to be dependent on CXCR4 ubiquitination, and does not involve stable interactions with TCR microclusters, as viewed at the nanoscale. Disruption of this process by mutation impairs CXCR4 contributions to cellular activation. Gαi2 undergoes active exclusion from the synapse, partitioning from centrally-accumulated CXCR4. Using a CRISPR-Cas9 knockout screen, we identify several diverse GPCRs with contributions to T cell activation, most significantly the sphingosine-1-phosphate receptor S1PR1, and the oxysterol receptor GPR183. These, and other GPCRs, undergo organization similar to CXCR4; including initial exclusion, centripetal transport, and lack of receptor-TCR interactions. These constitute the first observations of GPCR dynamics within the synapse, and give insights into how these receptors may contribute to T cell activation. The observation of broad GPCR contributions to T cell activation also opens the possibility that modulating GPCR expression in response to cell status or environment may directly regulate responsiveness to pMHC.

## Introduction

The adaptive immune system depends on the activation of antigen-specific lymphocytes to deliver an appropriate and coordinated response to infection or cellular dysfunction. Central to this are T cells, which express clonally unique T cell receptors (TCRs) capable of recognizing a restricted range of antigen-derived peptides presented by major histocompatibility (MHC) molecules on antigen-presenting cells (APCs), such as B cells and dendritic cells (DCs). The recognition of cognate peptide-MHC (pMHC) by TCR leads to activation of the T cell and formation of a large interface with the APC; in either the form of a stable immunological synapse (synapse) or a motile kinapse (Dustin, 2007; Mayya et al., 2018). This involves spatial organization into distinct zones that correspond to transitions in an underlying filamentous actin (F-actin) network, described as supramolecular activation clusters (SMACs): the central (c)SMAC corresponds to sparse F-actin bundles that enable access for bidirectional vesicular budding and fusion; the actinomyosin- and talin-rich peripheral (p)SMAC stabilizes adhesion; and the dendritic F-actin-rich distal (d)SMAC is an important site for signal initiation (Freiberg et al., 2002; Sims et al., 2007; Fritzsche et al., 2017). It is important to point out that part of the cSMAC includes a synaptic cleft into which TCR-enriched extracellular vesicles, including synaptic ectosomes and exosomes, soluble secreted proteins, and multiprotein complexes are released (Stinchcombe et al., 2001; Mittelbrunn et al., 2011; Choudhuri et al., 2014; Saliba et al., 2019; Bálint et al., 2020)—a process that occurs through the ramified actin network (Fritzsche et al., 2017). Kinapses are related to synapses by symmetry breaking with the dSMAC converting into a leading lamellipodium and pSMAC into a talin-rich focal zone (Smith et al., 2005; Sims et al., 2007). A common feature of both synapse and kinapse is F-actin-dependent TCR microclusters/protrusions that integrate with the larger actin network to influence synapse/kinapse balance (Varma et al., 2006; Beemiller et al., 2012; Kumari et al., 2015, 2020; Cai et al., 2017). Immunoglobulin superfamily, tumor necrosis factor/receptor families, and integrin family receptors—e.g., TCR, CD28, CTLA-4, PD1, CD40L, HVEM, LFA-1—are well-mapped in the immunological synapse including the recently described CD2 corolla (Demetriou et al., 2020). However, it is also evident that proteins from other families have significant contributions in this context, including members of the G protein-coupled receptor (GPCR) family. GPCRs are the largest family (>800 members) of cell surface receptors in the human genome and activate intracellular heterotrimeric G proteins and arrestins in response to extracellular ligand-binding (Rosenbaum et al., 2009). TCR-derived signals act in part through G-proteins (Stanners et al., 1995; Ngai et al., 2008) and arrestins (Fernández-Arenas et al., 2014), and are sensitive to factors under GPCR control, e.g., cAMP (Ledbetter et al., 1986; Abrahamsen et al., 2004). Several GPCRs have important regulatory function during T cell-APC communication, including receptors for lysophosphatidic acid (Oda et al., 2013), adenosine (Linnemann et al., 2009), adrenaline (Fan and Wang, 2009), and dopamine (Papa et al., 2017); however, the most ubiquitous are members of the chemokine receptor family.

Classically, chemokine receptors coordinate migration of T cells and other leukocytes between blood, lymphoid organs, and inflamed tissue by directing cells along localized chemokine gradients. Orthogonal CCL21 and CXCL10 gradients promote synapse breaking, whereas orthogonal CXCL12 and CCL5 gradients are generally permissive of synapse formation (Bromley et al., 2000). Consistent with this, signals from the TCR and chemokine receptors may be reciprocally regulated (Peacock and Jirik, 1999; Dar and Knechtle, 2007) and chemokine-mediated signaling in T cells is at least partially dependent on components of the TCR signaling system, e.g., Lck (Inngjerdingen et al., 2002), ZAP70 (Kremer et al., 2003), and the TCR itself (Newton et al., 2009). CXCR4 and CCR5 (Molon et al., 2005; Contento et al., 2008), as well as CCR7 (Laufer et al., 2018) are recruited to the synapse to act as coreceptors that enhance TCR-derived signals, increase synapse lifetime, and augment cytokine mRNA stability (Kremer et al., 2017). Such recruitment appears to be driven by TCR triggering which might also synergise with chemokine-driven receptor activation. Furthermore, direct physical association with TCR might be required for recruitment of CXCR4 (Kumar et al., 2006; Trampont et al., 2010) and CCR7 (Laufer et al., 2018). In the case of CXCR4 such association appears to be dependent on phosphorylation of Ser-339 by G protein-coupled receptor kinase-2 (GRK2) that is in turn activated by TCR-activated tyrosine kinases (Dinkel et al., 2018). However, delivery of CXCR4 into the synapse is also reportedly driven by the actin-binding protein drebrin, which by bridging CXCR4 to actin leads to its accumulation in the actin-rich regions of the synapse (Pérez-Martínez et al., 2010). Actin-enrichment is restricted to the periphery of the synapse, away from the major accumulations of TCR at the center, and hence would appear to be incompatible with simultaneous CXCR4 interaction with the TCR. Nonetheless, a C-terminally truncated form of CXCR4 associated with WHIM (warts, hypogammaglobulinemia, infections, myelokathexis) syndrome does not exhibit correct recruitment to and stabilization of the synapse (Kallikourdis et al., 2013), confirming the importance of this domain for CXCR4 coreceptor function. The spatiotemporal organization of CXCR4 and other GPCRs within the synapse has not been extensively studied, and the extent to which GPCRs can influence TCR signaling in the absence of ligation is poorly understood.

Alongside these considerations is the question of to what degree GPCR effects on T cell activation are dependent on signaling through associated G proteins. This is perhaps best characterized for Gαs-coupled GPCRs, such as the adenosine or adrenergic receptors, which increase local cAMP concentration through activation of adenylate cyclase. Ligand-dependent activation of Gαs activates the inhibitory kinase Csk in a cAMP-dependent manner (Vang et al., 2001), thereby inhibiting TCR signaling through ZAP70 (Linnemann et al., 2009) and downstream activation of integrins (Dimitrov et al., 2019). The contribution of Gαi-coupled signaling, which inhibits adenylate cyclase, is less well-understood. Many T cell-expressed GPCRs couple preferentially to Gαi proteins, including all chemokine receptors, and this signaling pathway is the primary driver of chemotaxis (Legler and Thelen, 2018). Several studies have reported chemokine-dependent effects on T cell activation that are sensitive to inhibition by pertussis toxin (PTx), which inactivates Gαi proteins (e.g., Bromley and Dustin, 2002; Smith et al., 2013). Nonetheless, upon recruitment to the synapse, chemokine receptors have also been observed to shift preference from Gαi- to Gαq/11-coupled pathways (Molon et al., 2005), which drive cell adhesion rather than migration (Mellado et al., 2001). However, Gαq is believed to be inhibited by active GRK2 (Mariggiò et al., 2006), and so it is not clear how much GPCRs within the synapse could promote Gαq signaling even if they are able to physically couple. Interpretation of experiments involving inhibition by PTx are also complicated by the observation that PTx activates the TCR signaling pathway to drive desensitization of chemokine receptors (Schneider et al., 2009), thereby impacting receptor effects beyond just Gαi-coupled processes. Alongside G protein signaling, chemokine receptors are sensitive to tyrosine-phosphorylation at a DRY motif at the cytoplasmic end of transmembrane helix 3 (Mellado et al., 1998), which is highly conserved across almost all GPCRs. Such phosphorylation can be mediated by Src-family kinases (Hauser et al., 2016), generating docking sites for SH2-domain containing proteins in a manner similar to the TCR itself and many tyrosine-based co-receptors.

In this study we use fluorescence microscopy techniques to examine the spatiotemporal organization of GPCRs within the synapse and identify the underlying molecular determinants. We focus primarily on the chemokine receptor CXCR4 due to its relative significance in T cell activation, and existence of previously published insights into its gross distribution in the synapse (Molon et al., 2005; Pérez-Martínez et al., 2010). In order to simulate T cell-APC interactions in an imaging-permissive manner we use planar supported lipid bilayers (SLBs) loaded with anti-CD3 Fab' to mimic TCR-pMHC engagement, and the recombinant integrin ligand ICAM1 (intercellular adhesion molecule one) to drive adhesion through binding to LFA1. This approach has been widely used in combination with total internal fluorescence microscopy (TIRFM) to visualize only the events occurring at the synapse (Calvo and Izquierdo, 2018). Through both ensemble imaging and single-particle tracking, we observe initial segregation of CXCR4 to the dSMAC, followed by active recruitment to the center over time. This is not due to physical TCR-CXCR4 interactions and is not sensitive to CXCR4 engagement of chemokine, coupling to G protein, or C-terminal/DRY motif phosphorylation, but may be dependent on ubiquitination. We report concomitant exclusion of the G protein Gαi2 from the synapse, which may offer an explanation for the cessation of Gαi-mediated signaling by chemokine receptors upon T cell activation. Finally, we assess the sensitivity of T cell activation to knockout of 28 diverse GPCRs and identify significant contributions for several receptors. Investigation of a subset of these GPCRs did not reveal clear correlation between intra-synapse organization and costimultory potential, but did suggest commonalities in receptor dynamics that may be applicable to many GPCRs.

## Results

### CXCR4 Undergoes Contact Time-Dependent Organization Within the T Cell Synapse

**Figure 1:**
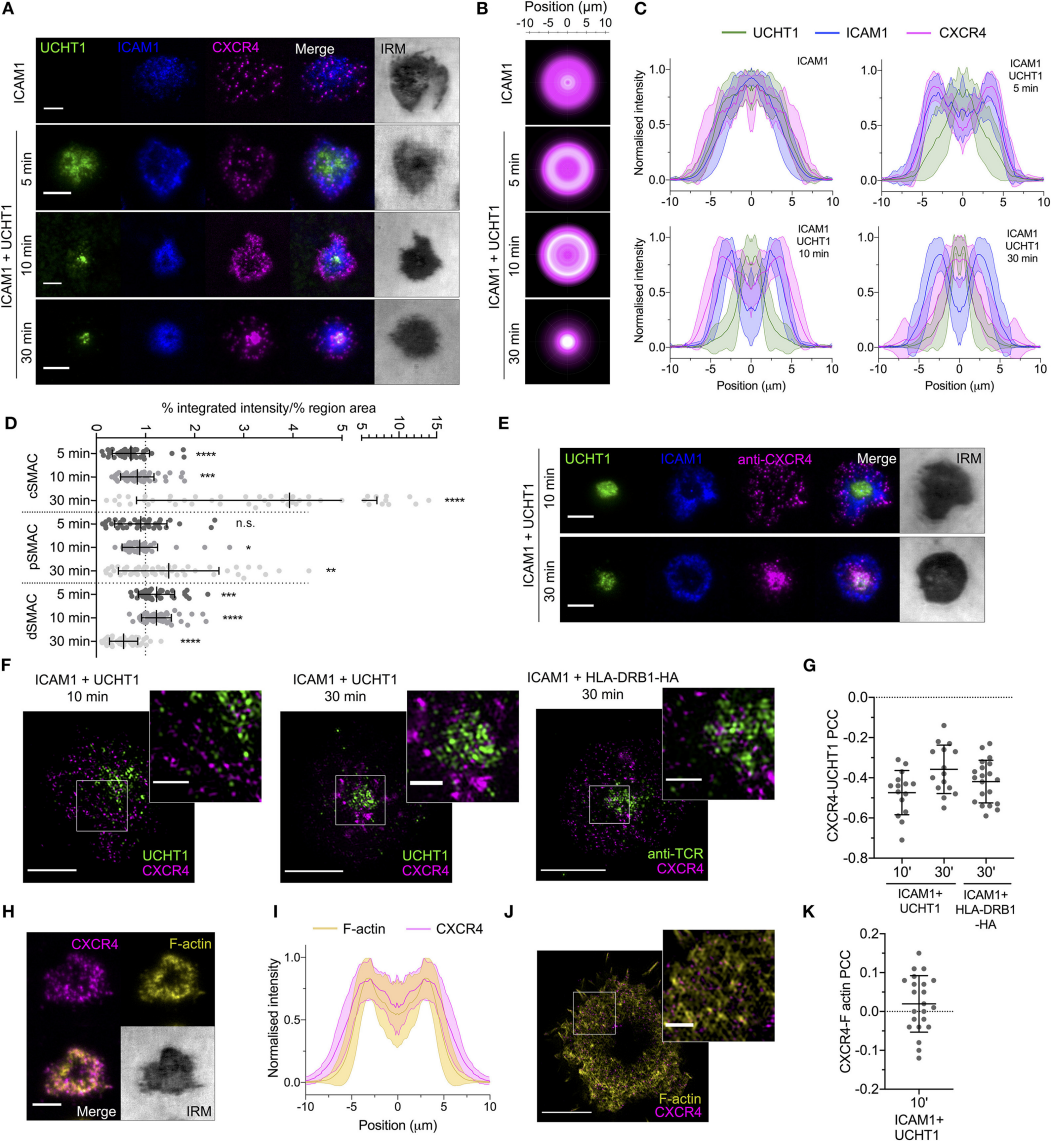
CXCR4 distribution within the synapse. **(A)** Representative TIRFM examples of CXCR4-HaloTag-expressing CD4^+^ T cell blasts interacting with non-activating (ICAM1) or activating (ICAM1 + UCHT1) SLB for 5–30 min. IRM, interference reflection microscopy. **(B)** Radial averages of CXCR4-HaloTag intensity from >30 cells for each indicated condition. **(C)** Cross-sectional normalized intensity profile of radial averages of all labeled proteins for each indicated condition. Plots are mean normalized intensity at each position ± std dev. **(D)** Relative enrichment of CXCR4-HaloTag intensity in cSMAC, pSMAC, and dSMAC regions of the synapse on activating SLB. Values are expressed as the percentage of total intensity within a region divided by the percentage of the total IRM-defined area that constitutes that region. A value of one indicates no relative enrichment or depletion from a region; >one indicates relative enrichment; <one relative depletion. Significance is shown relative to a value of one assessed with a one-sample, two-tailed *t*-test. Each point represents an individual cell; bars are mean ± std dev. **(E)** TIRF microscopy examples of anti-CXCR4-stained CD4^+^ T cell blasts interacting with activating SLB for 10 or 30 min. **(F)** TIRF-SIM examples of CXCR4-HaloTag-expressing CD4^+^ T cell blasts on SLBs presenting ICAM1 + UCHT1 Fab for 10 or 30 min (left & middle), or HA-restricted CD4^+^ T cell clone 40 on SLB presenting ICAM1 + HLA-DRB1-HA for 30 min. Inserts correspond to white boxes. **(G)** Pearson's correlation coefficient (PCC) values for CXCR4-HaloTag vs. UCHT1 in TIRF-SIM-imaged cells. **(H)** TIRF microscopy example of F-actin and CXCR4-HaloTag in a CD4+ T cell blast on activating SLB. **(I)** Normalized intensity profile for radially averaged F-actin and CXCR4-HaloTag signals in >30 cells. **(J)** TIRF-SIM example of F-actin and CXCR4-HaloTag on activating SLB for 10 min. **(K)** PCC values for CXCR4-HaloTag vs. F-actin in TIRF-SIM-imaged cells. All scale bars are 5 μm except for zoomed inserts (1 μm). *p < 0.05, **p < 0.01, ***p < 0.001, ****p < 0.0001, n.s., not significant. All pooled data represent a minimum of *n* = 3 independent donors.

We began by quantifying the spatiotemporal organization of CXCR4 within the synapse to determine how it relates to the various SMACs and their distinct signaling environments. Primary human CD4^+^ T cell blasts were transfected with mRNA encoding CXCR4 fused to a C-terminal HaloTag, then allowed to form contacts on SLBs presenting either ICAM1 alone at 200 molecules/μm^2^ (non-activating) or ICAM1 and anti-CD3 (UCHT1) Fab', at 200 and 30 molecules/μm^2^, respectively (activating). These were then imaged live at different time points using TIRFM, which visualizes only molecules within ~100 nm of the SLB. Whereas, CXCR4 distribution exhibited no obvious organization in contact with the non-activating SLB, CXCR4 exhibited a clear exclusion from the center of the contact within minutes on activating SLB, and from both the cSMAC and pSMAC in the mature synapse (Figures 1A–D; Supplementary Figure 1A). This distribution shifted over the lifetime of the synapse, with gradual enrichment of CXCR4 within the cSMAC clearly evident after 30 min (Figures 1A–D). Three-dimensional confocal microscopy revealed large amounts of CXCR4 away from the planar bilayer interface that could be consistent with receptor endocytosis, but also with presence in extracellular vesicles that accumulate between the cell and the SLB (Supplementary Figure 1B). Delivery of intracellular CXCR4 toward the synapse could also contribute to this observation. Staining of endogenous CXCR4 with fluorophore-conjugated anti-hCXCR4 antibody following fixation at 10 and 30 min yielded comparable observations (Figure 1E), indicating that at least some of the centrally accumulated CXCR4 remains at the cell surface or in extracellular vesicles. Late CXCR4 accumulation at the cSMAC was also evident in cells pre-stained with anti-hCXCR4 antibody before synapse formation (Supplementary Figure 1C), supporting the notion that CXCR4 in this region has been directly recruited from the plasma membrane.

Given the previous indications of a physical association between CXCR4 and the TCR (Kumar et al., 2006; Trampont et al., 2010), and that recruitment to the cSMAC would be an expected outcome of this, we examined the nanoscale organization of CXCR4 relative to TCR microclusters using TIRFM with structured illumination microscopy (SIM), which provides an effective isotropic resolution of ~100 nm. This was performed in fixed cells to avoid movement of molecules during image acquisition. CXCR4 exhibited marked segregation from TCR-enriched regions of the synapse both 10 and 30 min after exposure to the SLB (Figures 1F,G). To determine if recruitment of CD4 to TCR-pMHC complexes impacts possible CXCR4-TCR interactions, we repeated these experiments with a high-affinity T cell clone specific to peptide corresponding to influenza H3 haemagglutinin residues 338–355 bound to HLA-DRB1^*^09:01 (as used in Saliba et al., 2019), which was used to replace UCHT1 Fab' on the SLB at 30 molecules/μm^2^. CXCR4 in these cells underwent comparable organization to those activated with UCHT1 (Supplementary Figure 1D), and was similarly segregated from the TCR at the nanoscale (Figures 1F,G). These data, along with the different timing of TCR and CXCR4 accumulation in the cSMAC, argue against the formation of extensive stable CXCR4-TCR interactions.

Given the initial distal segregation of CXCR4, we examined whether this distribution correlated with well-described peripheral F-actin structures (Dustin and Cooper, 2000). In the mature, early (10 min) synapse, CXCR4 distribution correlated closely with that of F-actin stained with phalloidin (Figures 1H,I), in line with previous observations of CXCR4-actin connections in activated T cells (Pérez-Martínez et al., 2010). This organization was lost following acute inhibition of Src kinases or disruption of actin polymerisation (Supplementary Figure 1E), supporting the notion that CXCR4 redistribution depends on correct F-actin organization. TIRF-SIM imaging of CXCR4 relative to F-actin revealed no significant positive or negative Pearson correlation between the two (Figures 1J,K), indicating that CXCR4 is not associated with peripheral actin en masse, however this does not exclude the possibility of transient associations within individual receptors or the stable association of CXCR4 with filaments separate from the brightest actin structures that may not be readily detectable with imaging.

### CXCR4 Is Actively Recruited to the Center of the Synapse

**Figure 2:**
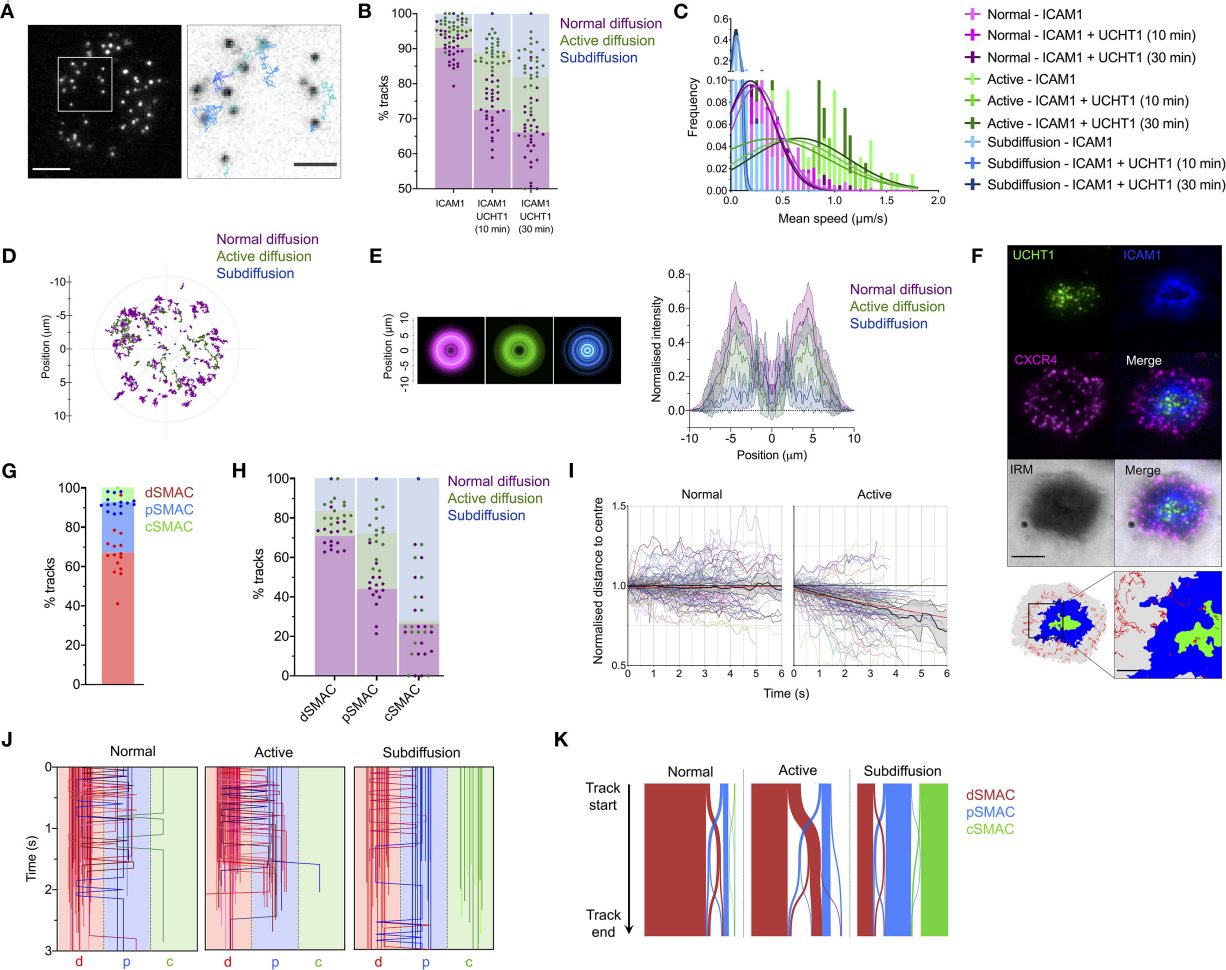
CXCR4 dynamics within the synapse. **(A)** Example of CXCR4-HaloTag single-particle tracking using TIRFM. The xy coordinates of all individual CXCR4-HaloTag spots were recorded every 50 ms and then linked together to derive particle tracks. **(B)** Proportion of single-particle CXCR4-HaloTag tracks exhibiting normal, active, or confined/subdiffusion in CD4^+^ blasts on inactivating or activating SLBs. Each point represents an individual cell. **(C)** Histogram of mean diffusion speed for tracks with different diffusive properties under activating and non-activating conditions. **(D)** Representative example showing relative location of different tracks within the synapse of a single cell. The entirety of all tracks over 30 frames in length are shown, centered around the approximate center of contact. **(E)** Radial averages of track locations across all cells imaged on activating SLBs (left), and cross-sectional normalized intensity profiles of those averages (right). Plots are mean normalized intensity at each position ± std dev. **(F)** Example of image partitioning based on IRM, ICAM1, and UCHT1 signals into d, p, and cSMAC regions, with single-particle tracks overlaid. **(G)** Proportion of all tracks that spend a minimum of three frames in the indicated regions. **(H)** Proportion of tracks exhibiting normal, active, or confined/subdiffusion in each indicated region. **(I)** Trajectories of 50 representative tracks undergoing normal or active diffusion, and mean ± 95% CL for all such tracks. Trajectories are expressed as normalized distance to center, in which the distance of the starting position of a track from the center of the imaged contact is given a value of one. Red lines indicate linear regression fit of all tracks. **(J)** Region position summaries of 50 example tracks for each diffusion type showing transitions between different regions of the synapse. Tracks are colored according to their starting region: red = dSMAC, blue = pSMAC, gree*n* = cSMAC. **(K)** Sankey plot summarizing starting region and transitions between regions for all recorded tracks. Bar width is proportional to the number of associated tracks within that diffusion category. All scale bars are 5 μm except for zoomed inserts (1 μm). All pooled data represent a minimum of *n* = 3 independent donors.

To assess how CXCR4 becomes enriched within the cSMAC, we examined the dynamic behavior of individual CXCR4 molecules through live TIRFM. Primary CD4^+^ T cell blasts transfected with low levels (200–2,000 molecules/cell) of CXCR4-HaloTag were imaged on SLBs containing ICAM1 alone or ICAM1 + UCHT1 Fab'. Videos were captured at 50 ms/frame for 15 seconds, then individual spots were identified and tracked over time in TIRFM to allow individual trajectories to be analyzed (Figure 2A; Supplementary Movie 1). Three forms of behavior were evident within the CXCR4 population: normal, unconstrained diffusion, active diffusion, and confined/subdiffusion. These three forms most likely correspond to receptors moving freely within the membrane (normal diffusion); receptors undergoing active transport through coupling to directional structures, e.g., actin-myosin (active diffusion); and receptors that are either immobile due to stable interactions with underlying structures or whose free diffusion is restricted to a highly confined area (subdiffusion). Under all conditions, the majority of molecules exhibited normal diffusion, however the proportion of receptors undergoing active and subdiffusion increased substantially when cells were activated with UCHT1 Fab' (Figure 2B; Supplementary Movie 2). Within the normal, active, and sub-diffusion populations there was no clear difference in behavior across different conditions (Figure 2C; Supplementary Figure 1F), indicating that cellular activation does not alter the characteristic diffusive modes, but simply changes their relative frequencies. The spatial distribution of different modes of diffusion varied markedly; with freely diffusing CXCR4 predominantly in the periphery of the contact, confined receptors more likely to be in the center (possibly in internal or extracellular vesicles), and actively diffusing receptors centrally offset relative to the majority of normally diffusing molecules (Figures 2D,E).

We next mapped absolute trajectory positions to regions of the synapse defined by UCHT1- or ICAM1-accumulation, and the IRM signal in single-frame images taken immediately before video acquisition (Figure 2F). As expected, the majority of receptors spent some time within the dSMAC, with the cSMAC containing the fewest tracks (Figure 2G). Within the dSMAC, the majority of receptors underwent free diffusion, whereas the cSMAC was occupied predominantly by subdiffusing receptors, and the pSMAC contained a substantial population of actively diffusing receptors (Figure 2H). On average, actively diffusing CXCR4 moved closer to the center of the synapse during the lifetime of the track, whereas freely diffusing receptors did not (Figure 2I). This indicates that CXCR4 actively migrates from the dSMAC toward the cSMAC, whereupon it becomes highly restricted and hence is retained. This is supported by the observation that only the actively diffusing receptors undergo substantial movement between the different areas of the synapse, predominantly from the dSMAC to pSMAC (Figures 2J,K). This indicates that the factors involved in the initial segregation of CXCR4 to the dSMAC also act as a barrier to passive CXCR4 entry into the pSMAC, but processes that progressively recruit CXCR4 to the cSMAC may overcome or circumvent this barrier. The fact that the majority of distal CXCR4 molecules undergo free diffusion supports the notion that although actin is a key driver of CXCR4 redistribution, this is not mediated by extensive, stable CXCR4-actin interaction.

### Ligation of CXCR4 Does Not Appreciably Impact Receptor Organization

**Figure 3:**
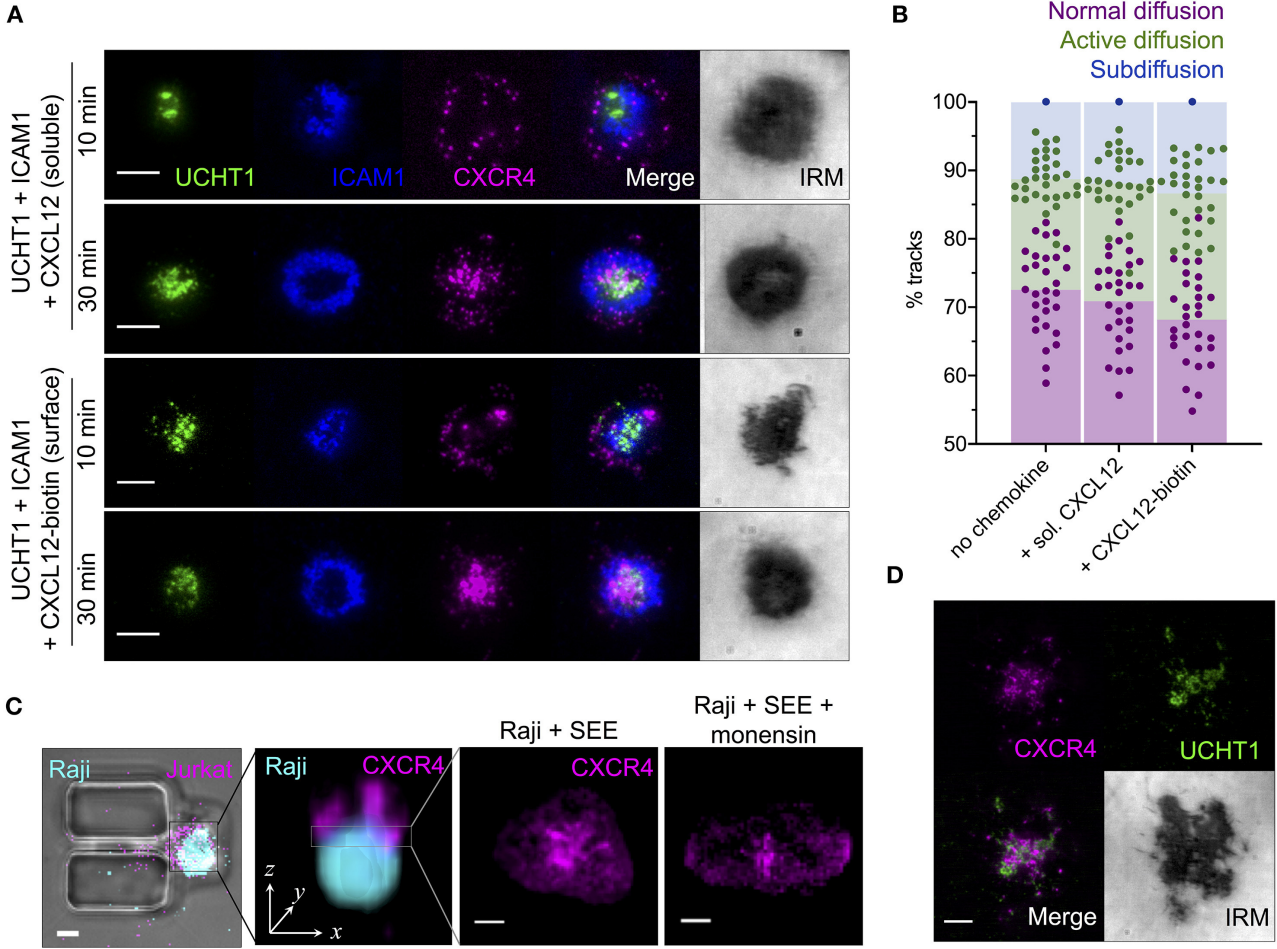
Effects of chemokine ligation upon CXCR4 distribution and dynamics. **(A)** TIRFM examples of CXCR4-HaloTag-expressing CD4^+^ T cell blasts interacting with activating SLB for 10 or 30 min in the presence of soluble or SLB-presented CXCL12. **(B)** Proportion of single-particle CXCR4-HaloTag tracks exhibiting normal, active, or confined/subdiffusion in cells on activating SLBs ± soluble/surface CXCL12 for 10 min. Each point represents an individual cell. **(C)** Example confocal microscopy image of a Raji-Jurkat conjugate within microfluidic vertical contact chamber (far left) and as three-dimensional z-stack (center left); then representative examples of CXCR4-HaloTag within the Raji-Jurkat interface in the presence or absence of protein transport inhibitor (monensin; right). **(D)** TIRFM example of CXCR4-HaloTag in a Jurkat E6.1 cell on activating SLB for 10 min. All scale bars are 5 μm. All pooled data represent a minimum of *n* = 3 independent donors.

All of the experiments described thus far were performed in the absence of CXCR4 ligation by chemokine. We therefore set out to determine how the observed organization of CXCR4 is influenced by its cognate ligand CXCL12, both in soluble and surface-presented forms. CXCR4-HaloTag-transfected primary human CD4^+^ T cell blasts were activated on SLBs as above, with the addition of either soluble CXCL12 at 0.1 μg/ml, or biotinylated CXCL12 attached to the SLB via a streptavidin linker at ~100 molecules/μm^2^. Interestingly, neither form of CXCL12 had any clear impact on CXCR4 distribution in either early or late synapses (Figure 3A), even though both promoted greatly increased cell migration on ICAM1-only containing SLBs (Supplementary Figure 1G). Single-molecule tracking of CXCR4-HaloTag in the early, mature synapse (10 min) revealed the same frequency of diffusion types (Figure 3B) and track characteristics within each diffusion type (Supplementary Figure 1H) regardless of which presentation of CXCL12 was present. This indicates a disconnect between the synaptic behavior of CXCR4 and its ligation state, in stark contrast to the situation within migrating T cells (Martínez-Muñoz et al., 2018).

Due to technical limitations of the SLB system, it was not possible to adequately replicate a scenario of CXCL12 release into the synapse by the APC, nor of potential differences in CXCL12 oligomerisation and/or activity through presentation by glycosaminoglycans and other chemokine-binding molecules on the APC. We therefore visualized CXCR4 distribution within the synapse of direct T cell-APC interactions. In order to permit high-resolution imaging in the x-y axial plane (as opposed to the z-axis orientation achieved through simple coculture), we employed a vertical-capture microfluidics approach (Jang et al., 2015) wherein APCs are first captured in holding pits within the microfluidics chamber and T cells flowed in afterwards to form a vertical conjugate (Figure 3C). This allowed confocal imaging of the conjugate synapse following fixation 30 min post T cell introduction. Due to the asynchronous way in which T cell-APC interactions begin in this system, it was not possible to precisely standardize synapse age prior to fixation, and hence imaging was restricted to the late synapse. Jurkat E6.1 cells expressing endogenous CXCR4 genomically fused to HaloTag were used in combination with Raji B cells loaded with Staphylococcal enterotoxin type E (SEE), which cross-links several common Vβ segment containing TCR to MHC class II molecules (Proft and Fraser, 2003). This maximized the likelihood of productive contact formation since all T cells were capable of responding to SEE-loaded B cells, and compensated for the reduced sensitivity of confocal vs. TIRFM as CXCR4-HaloTag expression was higher than in transfected primary cells. CXCR4 in these conjugates exhibited substantial central accumulation (Figure 3C) comparable to that observed in primary CD4^+^ T cells on SLBs, and also in Jurkat E6.1 cells on in the same system (Figure 3D). Incubation of Raji B cells with a monensin-containing protein transport inhibitor for 6 h prior to conjugate formation did not impact CXCR4 accumulation (Figure 3C), suggesting that this process is independent of active secretion into the synapse by the APC.

### CXCR4 Distribution Is Dependent on Ubiquitination in Its C-Terminal Domain

**Figure 4:**
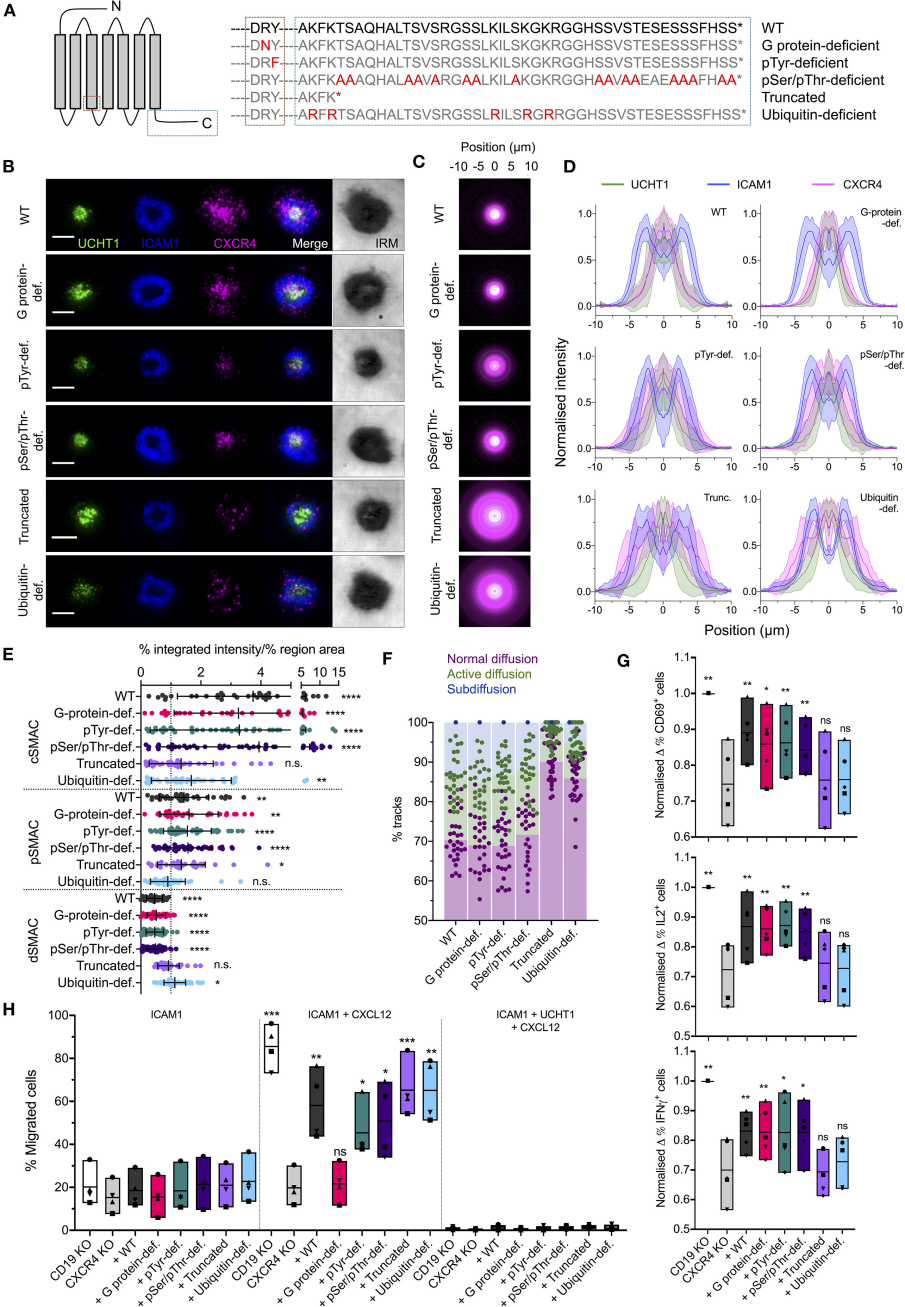
Organization of functional CXCR4 mutants in the synapse. **(A)** Summary of CXCR4 mutants used. **(B)** TIRFM examples of wild-type and mutant CXCR4-HaloTag in CD4^+^ T cell blasts on activating SLB for 30 min. Scale bars are 5 μm. **(C)** Radial averages of CXCR4-HaloTag intensity from > 30 cells for each mutant. **(D)** Cross-sectional normalized intensity profile of radial averages of all labeled proteins for each indicated condition. Plots are mean normalized intensity at each position ± std dev. **(E)** Relative enrichment of CXCR4-HaloTag mutant intensity in cSMAC, pSMAC, and dSMAC regions of the synapse on activating SLB. **(F)** Proportion of single-particle wild-type and mutant CXCR4-HaloTag tracks exhibiting normal, active, or confined/subdiffusion in cells on activating SLBs for 10 min. **(G)** Normalized change in CD69- (top), IL2- (middle), or IFNγ- (bottom) expressing cells upon incubation with anti-CD3/CD28 beads for 6 h. Cells are primary CD4^+^ blasts, KO for either CD19 or CXCR4 and transfected with indicated CXCR4 mutants. Each symbol represents a different T cell donor. Box plots show mean, minimum, and maximum values; significance is relative to untransfected CXCR4 KO cells as assessed by two-tailed *t*-test. **(H)** Percentage of KO, mutant-transfected cells that migrate across transwell inserts coated with ICAM1 or ICAM1+UCHT1 in the presence or absence of CXCL12 in the lower chamber. Each symbol represents a different T cell donor. Significance is relative to untransfected CXCR4 KO cells as assessed by two-tailed t test. *p < 0.05, **p < 0.01, ***p < 0.001, ****p < 0.0001, n.s., not significant. All pooled data represent a minimum of *n* = 3 independent donors.

To investigate the molecular determinants of CXCR4 organization within the synapse, we generated five function-specific C-terminally HaloTagged CXCR4 mutants and transfected them into primary CD4^+^ T (Figure 4A). These were: (1) deficient in G protein-coupling due to Arg-Asn substitution in the conserved DRY motif; (2) deficient in possible Tyr phosphorylation in the DRY motif due to a Try-Phe substitution; (3) deficient in all Ser/Thr phosphorylation in the C-terminal region due to substitution of all Ser/Thr residues with Ala; (4) C-terminally truncated after K314; and (5) deficient in C-terminal ubiquitination due to substitution of all Lys residues in the C-terminal domain with Arg. To avoid complicating factors from dimerisation with endogenous CXCR4, the native *CXCR4* gene was first disrupted in these cells by electroporation of an *in vitro*-generated ribonucleoprotein (RNP) complex consisting of the Cas9 nuclease and *CXCR4*-targetted guide RNA. CXCR4^−ve^ cells were isolated by fluorescence-activated cell sorting prior to transfection with CXCR4 mutants (Supplementary Figure 2A).

Importantly, whereas CXCR4 mutants in which G protein-coupling or possible Tyr phosphorylation at the DRY motif were inhibited showed wild type-like synapses (Figures 4B–E), truncation of the CXCR4 C-terminal region led to impairment of CXCR4 accumulation at the cSMAC. The latter replicates the observed aberrant CXCR4 accumulation observed on WHIM syndrome-associate truncated CXCR4 (Kallikourdis et al., 2013). Interestingly, this was not replicated by Ala substitution of Ser/Thr residues within the C-terminal region, which should impair phosphorylation by GRKs and interaction with arrestins, but was observed for mutant receptors in which potential sites of Lys ubiquitination were replaced with Arg (Figures 4B–E). This indicates a role for CXCR4 ubiquitination in the events orchestrating correct receptor migration within the synapse beyond the endpoint of internalization. Single particle tracking of mutant receptors in CXCR4^−ve^ cells showed diffusion behaviors correlating with this interfacial distribution. All forms of the receptor exhibited wild type-comparable normal, active, and sub diffusions at 10 min post activation except for the truncated and ubiquitin-deficient mutants, which underwent much less detectable active and subdiffusions (Figure 4F; Supplementary Movie 3).

### Correct CXCR4 Organization Is Required for Maximal T Cell Responses to Activation

To determine the impact of impaired CXCR4 organization upon its contribution to T cell activation, we stimulated CXCR4^−ve^ and *CD19*-targetted control primary CD4^+^ cell blasts with anti-CD3/anti-CD28 beads and assessed expression of CD69, IL2, and IFNγ 6 h post-stimulation using flow cytometry. CXCR4^−ve^ cells exhibited a moderate decrease in the fraction of cells positive for each of the three markers at 6 h (Figure 4G; Supplementary Figure 2C). Importantly, T cell activation could be partially restored to CXCR4^−ve^ cells through transfection of untagged wild type CXCR4, or of the G protein interaction-deficient, pTyr-deficient, or pSer/pThr-deficient mutants, but not of either C-terminally truncated CXCR4 or the ubquitination-deficient mutant (Figure 4G). Activation potential was not fully restored under any circumstances, however this may be due to the reduced expression of the transfected receptors compared to endogenous CXCR4 in wild type cells (Supplementary Figure 2B). Effects of mutant receptor expression upon CXCL12-induced chemotaxis were assessed using a transwell migration assay, wherein a gradient was generated between growth media containing 0 and 0.25 μg/ml CXCL12 separated by a 5 μm-pore transwell membrane, and the movement of cells up this gradient in 1 h quantified. All forms of the receptor restored responsiveness to CXCL12 in this assay, with the exception of the G protein interaction-deficient mutant, though none induced substantial migration across transwell inserts coated with UCHT1 (Figure 4H), in line with previous observations that CXCL12 does not override TCR signaling (Bromley et al., 2000). C-terminally truncated CXCR4 has previously been reported to sensitize cells to CXCL12 to overcome TCR-derived arrest signals (Kallikourdis et al., 2013), however this may not be replicated here again due to due to the relatively low expression of the transfected receptors.

### Gαi2 Undergoes Substantial Exclusion From the Synapse

**Figure 5:**
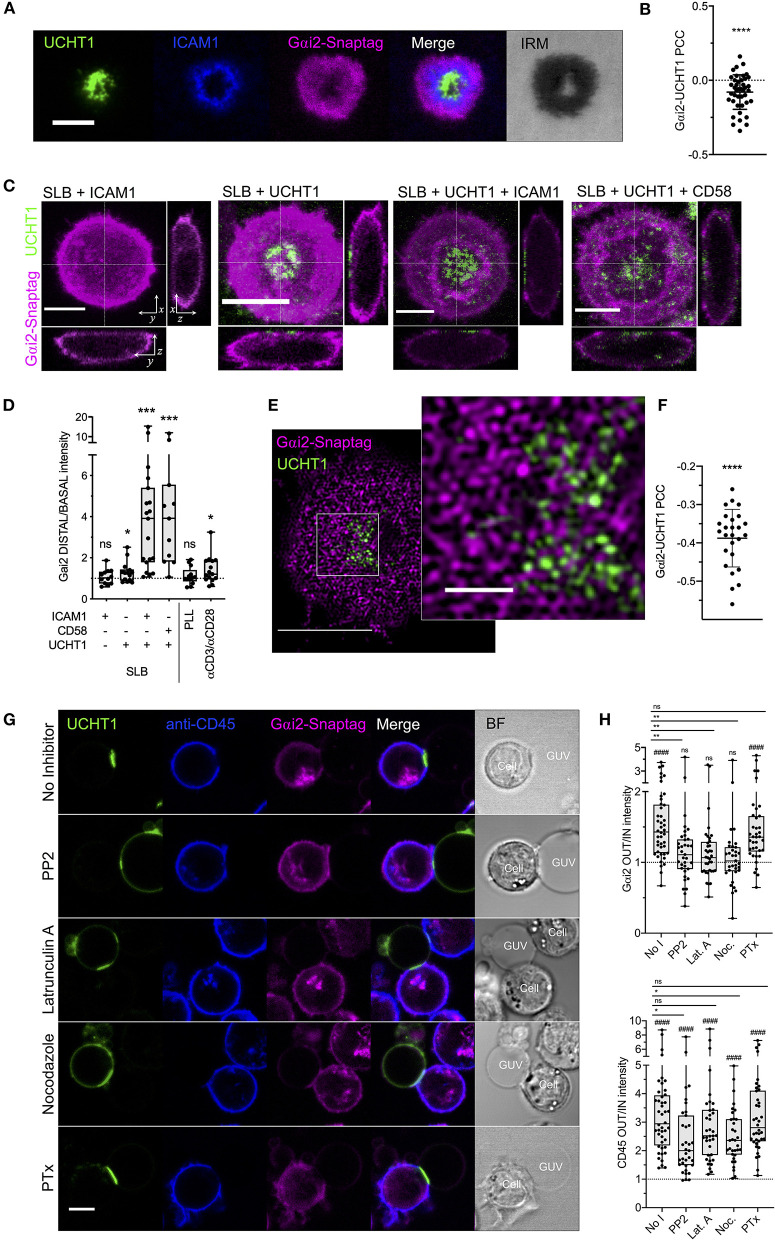
Gαi2 distribution within the synapse. **(A)** TIRFM example of Gαi2-SNAP-tag in a CD4^+^ T cell blast on activating SLB for 10 min. **(B)** PCC values for Gαi2 vs. UCHT1 in TIRF-imaged cells. Each point represents a single cell. Significance is shown relative to a value of 0 assessed with a one sample, two-tailed *t*-test. **(C)** Max-intensity projections and orthogonal views of confocal microscopy z-stacks of CD4^+^ T cell blasts on SLB presenting ICAM1, UCHT1, ICAM1 + UCHT1, or CD58 + UCHT1. **(D)** Ratio of Gαi2-SNAP-tag intensity at the distal vs. basal membranes for CD4^+^ T cell blasts on the indicated SLB compositions or glass surfaces coated with PLL or anti-CD3/CD28 antibodies. Box and whiskers show mean, maximum, minimum, upper, and lower quartiles. Significance is shown relative to a value of 1 assessed with a one-sample, two-tailed *t*-test. **(E)** TIRF-SIM example of Gαi2-SNAP-tag in a CD4^+^ T cell blast on activating SLB for 10 min. **(F)** PCC values for of Gαi2 vs. UCHT1 in TIRF-SIM-imaged cells. Significance is shown relative to a value of 0 assessed with a one sample, two-tailed *t*-test. **(G)** Confocal microscopy images at the equatorial plane of Gαi2-SNAP-tag-expressing, anti-CD45-stained CD4^+^ T cell blasts interacting with ICAM1 + UCHT1-bearing GUVs in the presence of indicated inhibitors. **(H)** Gαi2-SNAP-tag and CD45 intensities outside vs. inside cell-GUV contact regions. Significance vs. a fixed value of 1 as assessed by a one-sample two-tailed *t*-test is represented with hashes (####*p* < 0.0001). Significance between samples indicated by bars was assessed with a two-tailed *t*-test. *p < 0.05, **p < 0.01, ***p < 0.001, ****p < 0.0001, n.s., not significant. All scale bars are 5 μm except for zoomed inserts (1 μ m). All pooled data represent a minimum of *n* = 3 independent donors.

Our data so far indicate a disconnect between the behavior of CXCR4 within the synapse and its conventional coupling to G proteins of the Gαi family. CXCR4-CCR5 complexes are known to cease signaling via Gαi-dependent pathways upon formation of the synapse (Molon et al., 2005), however the reasons for this are poorly understood. While this inhibits CXCL12-dependent migration, it will also inhibit basal ligand-independent Gαi-coupled signaling exhibited by CXCR4 (Mona et al., 2016). We therefore chose to examine the synaptic distribution of the most abundant T cell-expressed Gαi protein, Gαi2 (Foley et al., 2010). Primary human CD4^+^ T cell blasts transfected with Gαi2 fused to SNAP-tag were examined with TIRFM on activating SLBs. Within fully formed synapses, Gαi2 underwent substantial redistribution to the dSMAC, with very clear negative correlation with TCR-UCHT1 distribution (Figures 5A,B; Supplementary Figure 3A). This redistribution of Gαi2 was evident during the early stages of IS formation before the cSMAC had fully coalesced (1-2 min; Supplementary Movie 4), indicating that this is not simply a product of molecular crowding, and did not appreciably change over the lifetime of the synapse (Supplementary Figure 3A). Using three-dimensional confocal microscopy, we observed that, relative to the rest of the cell, Gαi2 was substantially depleted across all but the extreme periphery of the contact in T cell blasts on activating SLB but not in resting cells on SLB containing ICAM1 alone (Figures 5C,D). Interestingly, T cell activation alone was not sufficient to drive maximal exclusion of Gαi2, as cells activated on SLBs containing only UCHT1 Fab' (Figures 5C,D) or on glass coated with anti-CD3/anti-CD28 antibodies (Figure 5D; Supplementary Figure 3B) exhibited much less Gαi2 depletion. To achieve maximal exclusion, adhesion molecules (either ICAM1 or CD58) and the formation of SMACs were also necessary. Non-specific cell adhesion and activation (Santos et al., 2018) on poly-_L_-lysine-coated glass did not induce Gαi2 exclusion (Figure 5D; Supplementary Figure 3B), indicating that both TCR triggering and engagement of either ICAM1 or CD58 are required for Gαi2 redistribution. TIRF-SIM of Gαi2 within the synapse revealed strong nanoscale exclusion from TCR-UCHT1-enriched domains (Figures 5E,F; Supplementary Figure 3C) even for the minority of residual Gαi2 within the cSMAC.

We next used giant unilamellar vesicles (GUVs) to activate Gαi2-SNAP-tag-transfected CD4^+^ T cell blasts and observed the distribution of Gαi2 with confocal microscopy. GUVs are analogous to SLBs except that they exist as spherical vesicles 10–100 μm in diameter, which can be loaded with His-tagged proteins via Ni-NTA-functionalised lipids (Jenkins et al., 2018). This allows x-y cross-sectional images to be captured at the equatorial plane of T cell-GUV contacts that is not possible with the SLB approach. As expected, Gαi2 was largely excluded from contacts between T cell blasts and GUVs presenting UCHT1 Fab' and ICAM1 (Figures 5G,H). CD45, a classical example of IS-excluded molecules (Dustin, 2014), was also excluded, whereas UCHT1 was enriched in the contact. Disruption of the synapse 15 min after formation by acute addition of inhibitors of Src kinase activity (PP2), or polymerisation of actin (latrunculin A) or microtubules (nocodazole) led to a loss of Gαi2 exclusion from the contact even though CD45 exclusion was still evident (Figures 5G,H). 18 h pre-treatment with PTx, which inhibits Gαi activity and coupling to GPCRs, did not impair Gαi2 exclusion. These data indicate that the redistribution of Gαi2 upon formation of the synapse is dependent on active cytoskeletal processes and continuous TCR signaling, and not upon active coupling to GPCRs. This opens the possibility that the inversely directional movement of CXCR4 and Gαi2 may be a deliberate mechanism by T cells to prevent CXCR4-Gαi coupling in response to TCR triggering, and hence to dampen pro-migratory CXCR4 signaling.

### Numerous GPCRs Exhibit Modulatory Functions on T Cell Activation

Since Gαi-coupled signaling is a common pathway for many T cell-expressed GPCRs, we questioned whether many such receptors might experience altered signaling within the synapse due to the redistribution of Gαi2. Modulatory function in T cell responses has been reported for several GPCRs (e.g., Contento et al., 2008; Linnemann et al., 2009; Oda et al., 2013; Laufer et al., 2018), however in most cases this has been examined in the context of receptor ligation rather than inherent ligand-independent activity, and no exhaustive screen of GPCR contributions to T cell activation has thus far been performed. We therefore set out to determine which, if any, GPCRs commonly expressed in CD4^+^ T cells influenced cellular responses to activation in the absence of exogenous receptor ligation. Using publicly available whole genome RNA sequencing (RNA-seq) data from the BluePrint consortium (Expression Atlas: E-MTAB-3827) we identified all GPCRs expressed to a level above five fragments per kilobase exon per million reads mapped (FPKM) in either primary total or effector memory CD4^+^ T cells. This identified 28 GPCRs, the majority of which were members of the *Rhodopsin* family, with many known to couple to Gαi/o family members (Supplementary Table 2). The highest FPKM belonged to CXCR4, however many other receptors also exhibited strong expression. This panel of receptors did not include a number of known influencers of T cell activation, including adenosine (Linnemann et al., 2009) and adrenergic (Fan and Wang, 2009) receptors, most likely because they are not highly abundant at the mRNA level or are inconsistently expressed. Although the ligand-dependent effects of these receptors are well-reported, we chose not to pursue them here as their low copy number reduced the likelihood of inherent ligand-independent effects. We cannot, however, exclude the possibility of ligand-independent effects of low-transcript GPCRs not investigated here.

Using the Cas9 RNP approach described above, the genes encoding candidate receptors, as well as those encoding CD3δ and CD28, were individually disrupted in resting human CD4^+^ T cells isolated from blood (guide sequences given in Supplementary Table 2). These were then divided into two populations, one of which was kept in resting culture without additional IL2, and the other was blasted for 3 days with anti-CD3/anti-CD28 beads and cultured in the presence of 100 U/ml IL2. Seven days post-transfection, all cells were activated either with anti-CD3/anti-CD28 beads or in co-culture with donor-matched APCs loaded with titrated amounts of SEE. For the activation of resting cells (which we consider to be predominantly naïve given extended culture without IL2, selecting against resting effector cells), activated monocyte-derived DCs (moDCs) were used as APCs, whereas for blasted cells B cells were used. Expression of CD69, IL2, and (for blasted cells only) IFNγ 6 h post-activation was assessed using flow cytometry (Supplementary Figure 4A) and normalized to the response observed in control cells transfected with RNP complexes targeting *CD19*. Cytokine retention was enhanced by the addition of a monensin-containing protein transport inhibitor 2 h after the start of activation. The efficacy of gene disruption was confirmed through TIDE (Tracking of Indels by DEcomposition) analysis of genomic DNA isolated from blasted cells 7 days post-transfection (Brinkman et al., 2014). This reliably reported approximate disruption efficiency for both the blasted and resting populations (Supplementary Figures 4B–D). Cells were not selected for receptor knockout, so the cells used in stimulation experiments represented a population of majority homozygous knockout with a minority of wild type and heterozygous partial knockouts.

**Figure 6:**
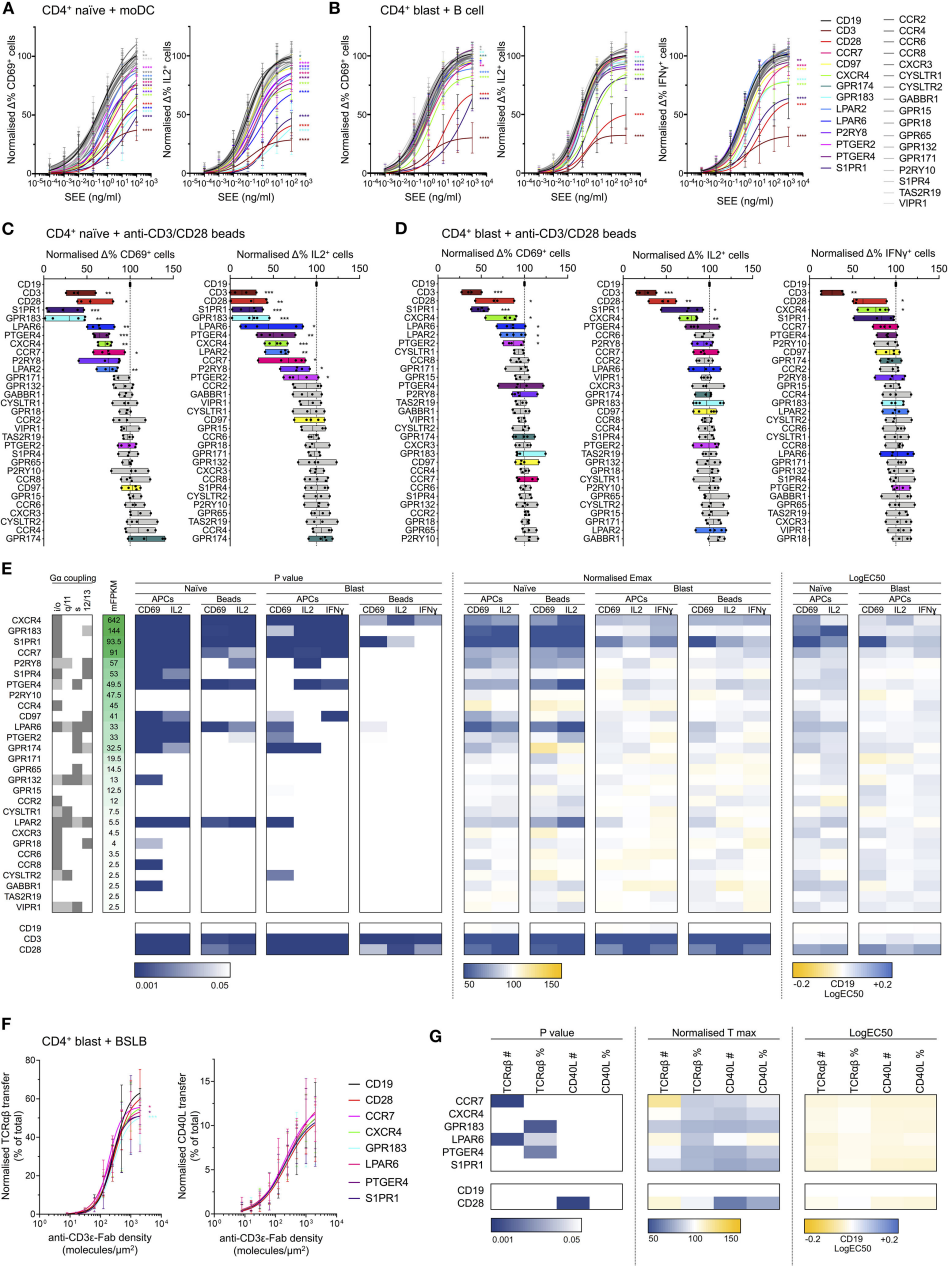
Effects of GPCR knockout in activation of naïve and blasted CD4^+^ T cells. **(A)** Normalized change in CD69^+^ (left) and IL2^+^ (right) naïve CD4^+^ T cells incubated with moDCs loaded with titrated concentrations of SEE. Plots show mean ± std dev., with best-fit non-linear response curves for each target. Datasets are colored according to target (key far right), with non-gray sets used only for targets exhibiting significant effects in both the CD69 and IL2 responses. Significance relative to the CD19 target data was assessed using an extra sum-of-squares F test and is indicated for all significant (*p* < 0.05) datasets. **(B)** Normalized change in CD69^+^ (left), IL2^+^ (center), and IFNγ (right) blasted CD4^+^ T cells incubated with B cells loaded with titrated concentrations of SEE. Data are represented as in **A**. **(C)** Normalized change in CD69^+^ (left) and IL2^+^ (right) naïve CD4^+^ T cells incubated with anti-CD3/CD28 beads. Boxes show mean, minimum, and maximum values, significance is shown relative to CD19 as assessed with a two-tailed *t*-test. Non-gray datasets are colored as in **A**. Each point represents a different T cell donor. **(D)** Normalized change in CD69^+^ (left), IL2^+^ (center), and IFNγ (right) blasted CD4^+^ T cells incubated with anti-CD3/CD28 beads. Data are represented as in **C**. **(E)** Summarized GPCR knockout screen data. Calculated F test *p*-values, normalized Emax, and logEC50 for all assays are represented as a heatmap, colored according to the corresponding scales below. For T cell-APC assays, Emax was derived as the value of the fitted response curve at the highest SEE concentration. Reported G protein coupling for each GPCR is shown as dark gray (primary coupling), light gray (secondary coupling), or white (no coupling), as listed in the GPCR database (gpcrdb.org). Receptors are ordered according to mean FPKM (mFPKM) values in RNA-seq from primary total or effector memory CD4^+^ T cells (E-MTAB-3827). **(F)** Normalized transfer of TCRαβ (left) or CD40L (right) from CD4^+^ blasts to BSLBs presenting ICAM1, CD40, and titrated densities of UCHT1, as a percentage of total cellular TCRαβ/CD40L. Data are represented as in **A**. **(G)** Summarized BSLB transfer assay data. Calculated F test p values, normalized Tmax, and logEC50 for all assays are represented as a heatmap, colored according to the corresponding scales below. Values are shown for raw amount of protein transferred (TCRαβ# or CD40L#) or as a percentage of total cellular protein (TCRαβ% or CD40L%). *p < 0.05, **p < 0.01, ***p < 0.001, ****p < 0.0001. All pooled data represent a minimum of *n* = 4 independent donors.

Knockout of several GPCRs had a significant effect on T cell responses to activation by SEE-loaded APCs (Figures 6A,B,E). This was most strongly evident in the naïve CD4^+^ population, wherein disruption of 12 GPCR genes significantly altered all measured responses, compared to four in blasted T cells (Figure 6E). This is perhaps unsurprising given the increased dependence of naïve T cells for costimulation during activation compared to effector cells. The genes with the greatest effects on responses to activation were typically those with the greatest transcript abundance in the RNA-seq data (Figure 6E), most substantially *CXCR4, GPR183, S1PR1, CCR7, P2RY8, PTGER4*, and *LPAR6*. This correlation was not absolute, however, as disruption of *LPAR2* also exhibited effects on response to activation despite having only a low associated FPKM. Similarly, several genes with relatively high associated FPKM values exhibited no clear effect, including *P2RY10* and *CCR4*.

Although several receptors only appeared to influence responses in naïve cells, this was most striking for GPR183, which had a very significant effect in naïve cells but no clear effect in blasted cells. Indeed, knockout of both GPR183 and S1PR1 had an unexpectedly dramatic impact on naïve T cell responses, with a greater loss of response than for knockout of CD28. The EC50 values relative to SEE concentration for GPR183 and S1PR1 knockouts were 1–2 orders of magnitude greater than the control cells, suggesting a possible central role in signal amplification from the TCR and/or CD28.

Responses to activation with anti-CD3/anti-CD28 beads were typically less sensitive to GPCR knockout than that with SEE-loaded APCs (Figures 6C,D,E). In blasted cells, only CXCR4 exhibited a consistent contribution to all three activation markers, with S1PR1 also having a significant effect on CD69 and IL2 responses. Naïve cells again showed greater sensitivity to GPCR knockout, though of the 12 receptors with consistent contributions in APC-mediated activation, five (P2RY8, S1PR4, CD97, PTGER2, and GPR174) failed to exhibit significant effects upon CD69 and/or IL2 responses following bead-mediated activation.

In all cases, the effects observed were not due to altered expression of either TCR or CD28 in the knockout cells, as these were unaffected by GPCR disruption (Supplementary Figure 4E). The only evident difference in the resting state of any knockout cells was the basal CD69 expression in S1PR1-deficient cells, which was greatly enhanced relative to all other cells (Supplementary Figures 4E,F). This is not unexpected since S1PR1 and CD69 undergo reciprocal negative regulation due to direct physical interactions (Bankovich et al., 2010). The effects of S1PR1-knockout on CD69 responses are therefore more difficult to interpret, however the fact that effects were also observed for IL2 and IFNγ responses increases confidence that these effects are genuine.

To examine the potential impact of GPCR knockouts on T cell effector function, we quantified release of CD40L- and TCRαβ-containing synaptic ectosomes from CD4^+^ blasts. Following disruption of *CD28, CCR7, CXCR4, GPR183, LPAR6, PTGER4, S1PR1*, or *CD19*, CD4^+^ blasts were incubated with bead-supported lipid bilayers (BSLBs) presenting ICAM1 at 200 molecules/μm^2^, CD40 at 20 molecules/μm^2^, and UCHT1 Fab' at titrated densities from 0 to 2,000 molecules/μm^2^ (Saliba et al., 2019). These are equivalent to SLBs but formed around silica beads, allowing transferred proteins to be retained and quantified. After 90 min BSLBs were detached from cells, stained for CD40L and TCRαβ and assessed with flow cytometry. Among GPCR and CD28 knockouts, no significant differences were observed in the transfer of synaptic ectosomes containing CD40L and TCRαβ to BSLBs, indicating that these had no participation in the delivery of helping factors by CD4^+^ T cells (Figures 6F,G; Supplementary Figure 4G).

### GPCR Dynamics Do Not Correlate With Costimulatory Potential

**Figure 7:**
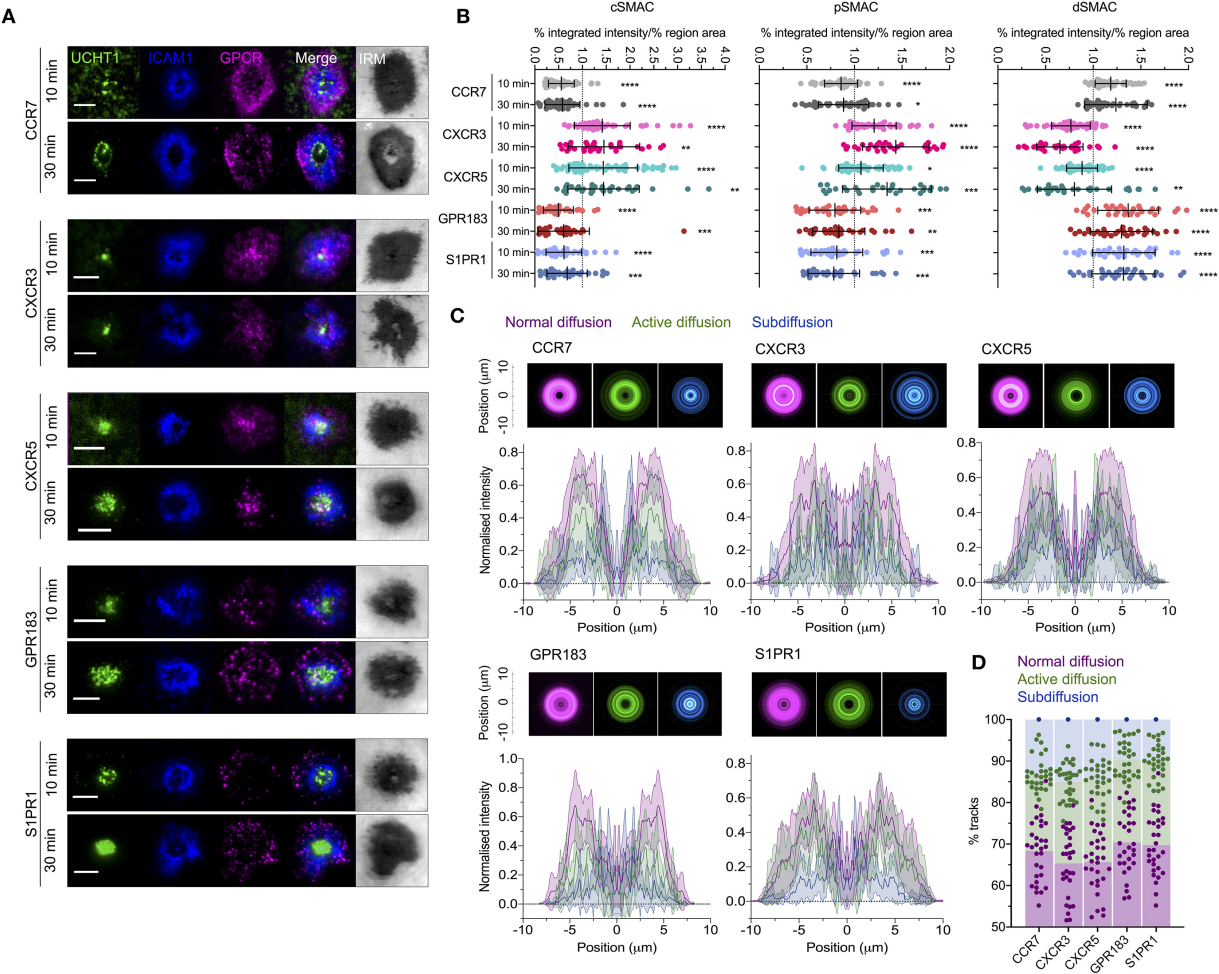
Distribution and dynamics of additional GPCRs within the synapse. **(A)** Representative TIRFM examples of HaloTag-fused GPCRs in transfected CD4^+^ T cell blasts interacting activating (ICAM1 + UCHT1) SLB for 10 or 30 min. **(B)** Relative enrichment of GPCR-HaloTag intensities in cSMAC, pSMAC, and dSMAC regions of the synapse on activating SLB. Values are expressed as the percentage of total intensity within a region divided by the percentage of the total IRM-defined area that constitutes that region. Significance is shown relative to a value of 1 assessed with a one-sample, two-tailed *t*-test. Each point represents an individual cell; bars are mean ± std dev. **(C)** Radial averages of single-particle track locations for all GPCRs and all diffusion types in CD4^+^ blasts on activating SLBs for 10 min (top), and cross-sectional normalized intensity profiles of those averages (bottom). Plots are mean normalized intensity at each position ± std dev. **(D)** Proportion of different GPCR-HaloTag tracks exhibiting normal, active, or confined/subdiffusion in CD4^+^ blasts on activating SLBs for 10 min. All scale bars are 5 μm except for zoomed inserts (1 μ m). All pooled data represent a minimum of *n* = 3 independent donors. *p > 0.05, **p > 0.01, ***p > 0.001, ****p > 0.0001.

**Figure 8:**
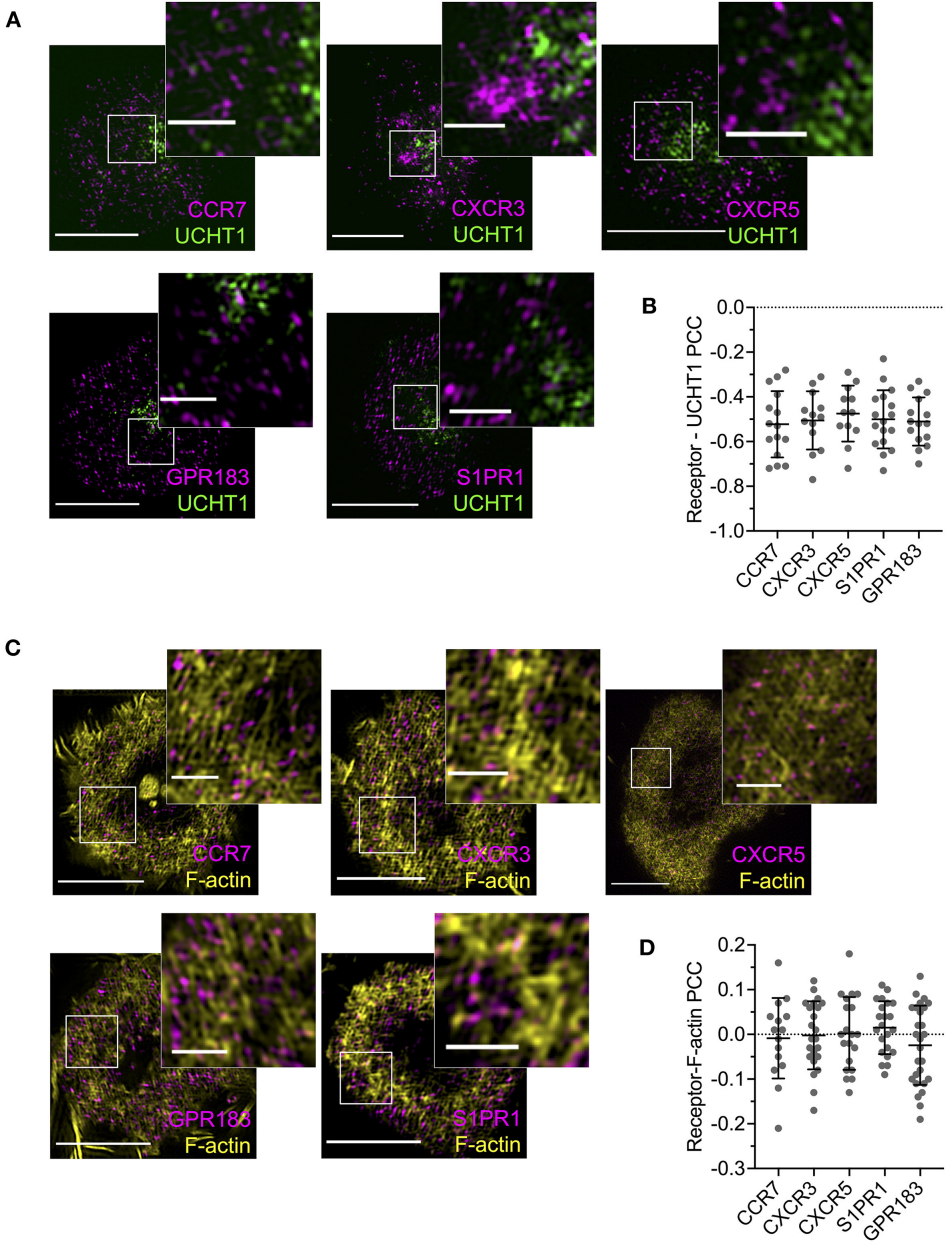
Nanoscale distribution of additional GPCRs. **(A)** TIRF-SIM examples of GPCR-HaloTag-expressing CD4^+^ T cell blasts on activating SLBs for 10 min. Inserts correspond to white boxes. **(B)** Pearson's correlation coefficient (PCC) values for GPCR-HaloTag constructs vs. UCHT1 in TIRF-SIM-imaged cells. **(C)** TIRF microscopy examples of F-actin and GPCR-HaloTag constructs in CD4+ T cell blasts on activating SLB for 10 min. **(D)** Pearson's correlation coefficient (PCC) values for GPCR-HaloTag constructs vs. F-actin in TIRF-SIM-imaged cells. All scale bars are 5 μm except for zoomed inserts (1 μm). All pooled data represent a minimum of *n* = 3 independent donors.

Given the evident effects of several tested GPCRs on T cell responses, we examined the distribution and dynamics of a subset with the aim of identifying any commonalities with CXCR4. We chose two receptors that showed costimulatory function in both blast and naïve cells (CCR7 and S1PR1), one that had an effect only in naïve cells (GPR183), one that had no evident effect (CXCR3), and one that is not typically expressed in conventional T cells (CXCR5—normally restricted to follicular helper T cells). All five receptors were transfected as C-terminal HaloTag fusions into blasted primary CD4^+^ T cells and assessed by TIRFM on activation SLB. CCR7, GPR183, and S1PR1 exhibited distribution at 10 min that closely resembled that of CXCR4—with substantial depletion from the central regions of the synapse and enrichment in the dSMAC (Figures 7A,B). Conversely, CXCR3 and CXCR5 showed much greater accumulation in the cSMAC at 10 min, but less extensively so than CXCR4 at 30 min. The distribution of all receptors remained broadly unchanged between 10 and 30 min (Figures 7A,B), in stark contrast to CXCR4. Interestingly, despite the two different overall distributions across the receptors, when assessed by single-particle tracking all five demonstrated comparable dynamics and spatial distribution of diffusion types (Figures 7C,D; Supplementary Movie 5). This was highly comparable to that observed for CXCR4, with a majority of freely diffusing tracks that were restricted largely to the distal regions of the synapse; actively diffusing tracks moving centrally; and tracks undergoing subdiffusion predominantly in the cSMAC. When investigated by TIRF-SIM, all five receptors exhibited segregation from regions of TCR enrichment (Figures 8A,B), and no detectable nanoscale correlation with F-actin (Figures 8C,D), again in line with the organization observed for CXCR4. There was no evident correlation between receptor dynamics or nanoscale organization and reported impact on T activation. Given this common behavior, it seems likely that the observed differences in gross receptor distribution (Figures 7A,B) are the result of differences in concurrent receptor trafficking—i.e., internalization from or endocytic deliver to the synapse.

## Discussion

**Figure 9:**
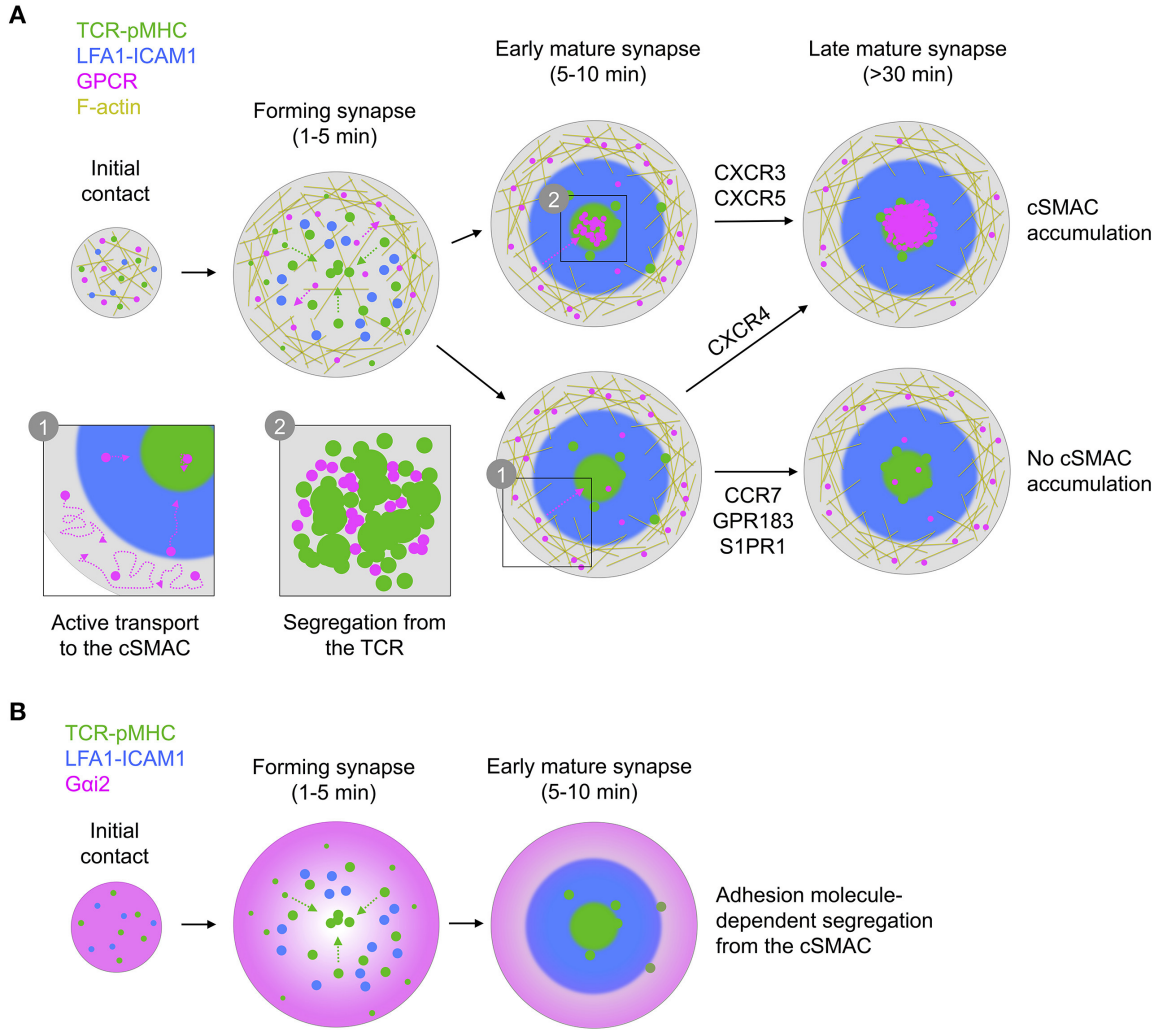
Graphical summary of receptor/G protein distribution and dynamics. **(A)** All GPCRs examined exhibit similar dynamics, characterized by free diffusion at the periphery of the synapse with active transport to the center. This leads to different overall distribution, with either no, early, or late accumulation in the cSMAC, dependent on receptor. In all cases, receptors in the cSMAC are segregated from TCR clusters. **(B)** Gαi2 is depleted from the center of the contact even before full formation of the cSMAC, with this becoming more pronounced during maturation of the synapse.

In this study we observe that CXCR4, as a key GPCR of interest, undergoes active reorganization within the synapse, characterized by initial exclusion to the periphery followed by active transport toward the center (Figure 9A). The correlation of CXCR4 with F-actin-enriched regions is consistent with a previous report of CXCR4-drebrin-actin interactions upon TCR triggering (Pérez-Martínez et al., 2010), however our observation of freely diffusing CXCR4 in these regions indicates that such interactions are likely not sufficiently stable to fully restrict receptor movement. We do not observe nanoscale correlation of CXCR4 (or indeed any GPCR here studied) with the TCR, arguing against the formation of stable CXCR4-TCR complexes. Previous reports of such complexes have been based primarily on resonance energy transfer experiments or diffraction-limited imaging (Kumar et al., 2006; Trampont et al., 2010), which could also be consistent with increased crowding of CXCR4 and TCR in the cSMAC without the need for direct interaction. Nonetheless, we cannot exclude the possibility of short-lived interactions that transiently impact signaling during microcluster migration. We also cannot comment on how CXCR4-TCR distribution may vary according to TCR-pMHC stability, and it is possible that stable complexes may be induced by TCRs of a particular affinity.

Using single-particle tracking, we observed substantial cell activation-dependent changes in CXCR4 dynamics characterized by a large fraction of freely diffusing receptors with smaller populations of actively migrating and subdiffusing molecules. Such behavior is similar to that of the TCR, except that we observed no obvious formation of migrating CXCR4 microclusters, and that the time scale to accumulation in the cSMAC was much slower than that of the TCR. The majority of normally diffusing CXCR4 is in contrast to a previous study reporting only ~11% of freely diffusing receptors on fibronectin-coated glass (Martínez-Muñoz et al., 2018). This difference could arise from distinct behavior of CXCR4 on immobile fibronectin vs. mobile ICAM1. In both studies the majority of tracked receptors remained mobile, but on fibronectin ~78% of these remained within 200 nm over the >2 s life of the track. A marginal increase in CXCR4 mobility on ICAM1 may have allowed this large fraction of receptors to exhibit normal diffusion over the >1.5 s track length acquired in this study.

Although comparisons of our single-particle diffusion data across conditions is valid, certain caveats should be considered when directly interpreting frequencies of different species. Due to the nature of diffraction-limited imaging, clustered receptors will be underreported in the tracking data as they will be detected as single spots. In the case of CXCR4, this seems most likely for subdiffusing receptors near the center of the synapse. Similarly, new fast-diffusing spots are more likely to enter the imaging field during the course of image capture, again causing under-representation of slow-moving spots in the data. Conversely, faster moving spots are more likely to leave the imaging field within 30 frames, and are more difficult to accurately connect, which will reduce their representation in the reported tracks. These effects mean the absolute receptor proportions described herein should be interpreted with care.

We failed to detect any significant effects of CXCL12 ligation on CXCR4 organization or dynamics. This was surprising given the effects of CXCL12 on CXCR4 ubiquitination (Marchese and Benovic, 2001) and internalization (Haribabu et al., 1997), however it appears that TCR signaling supersedes the ordinary effects of CXCL12, as previously described (Bromley et al., 2000). More unexpected was the possible dependence on CXCR4 ubiquitination. Previous studies have suggested that regulation of CXCR4 behavior in the synapse is mediated by GRK-dependent phosphorylation of the C-terminus (Dinkel et al., 2018) or on association with arrestins (Fernández-Arenas et al., 2014). We failed to see clear effects of C-terminal Ser/Thr-Ala substitution (which will also block arrestin binding) on either CXCR4 dynamics or overall organization, or on its costimulatory potential. CXCR4 distal from the T cell-APC contact has previously been observed to be redirected to the synapse in an arrestin-dependent manner (Fernández-Arenas et al., 2014), which we did not assess, opening the possibility that CXCR4 undergoes first arrestin- then ubiquitin-dependent regulation at different stages of its delivery and organization. This is particularly interesting since CXCR4 ubiquitination is partially dependent on phosphorylation of the C-terminal domain (Marchese and Benovic, 2001), which raises the question of why mutation of phosphorylation sites did not have the same effect as mutation of ubiquitination sites. It is still possible that such mutation affects CXCR4 behavior in a way that we were not examining. Moreover, it is possible that C-terminal Lys-Arg substitution has impacts independent of ubiquitin. Nonetheless, the ability of both the C-terminally truncated and ubiquitin-deficient mutants to restore migration but not full responsiveness to activation indicates that the reduction of activation in CXCR4^−ve^ cells is not a product of reduced cell mobility.

Ubiquitination of GPCRs is subject to complex, receptor-specific regulation (Kennedy and Marchese, 2015), and although most commonly described in the context of receptor internalization, it is not a pre-requisite for GPCR removal from the plasma membrane (Kang et al., 2014). Ubiquitination of CXCR4 is required for sorting into intraluminal vesicles via the ESCRT (endosomal sorting complex required for transport) pathway (Marchese, 2014), which also regulates the sorting of proteins into synaptic ectosomes (Saliba et al., 2019). Irregular receptor accumulation of ubiquitin-deficient and C-terminally truncated CXCR4 could emerge from defects in correct receptor trafficking, through endocytosis and/or incorporation into synaptic vesicles, though it is not clear how this impacts CXCR4 signaling. It is well established that GPCR signaling does not immediately terminate upon endocytosis (Weinberg and Puthenveedu, 2019), and removal of CXCR4 from the synaptic plasma membrane could serve to prevent G protein-mediated signaling while maintaining signaling via arrestins or other partners. Arrestins help coordinate correct TCR trafficking to and from the synapse (Fernández-Arenas et al., 2014) and so endocytosed CXCR4 (or other GPCRs) may contribute to this regulation. Alongside endocytosis, T cells release substantial numbers of synaptic vesicles (Choudhuri et al., 2014), and it is possible that CXCR4 or other GPCRs are incorporated. Previous proteomic analysis of the composition of synaptic vesicles only identified CD97 as the sole GPCR enriched in such vesicles (Saliba et al., 2019), however this does not preclude incorporation of others under different circumstances. GPCRs can be incorporated into extracellular vesicles at cilia (Nager et al., 2017), which share many close similarities with the immunological synapse (Cassioli and Baldari, 2019), so such a process in activated T cells is not implausible. In such a case, CXCR4 release would likely serve to terminate migratory signaling and possibly to act as local scavengers of chemokine ligands. Our observations can be explained without the need for CXCR4 release in vesicles, and the lack of correlation with the TCR may argue against this, however their enrichment at the cSMAC would bring them into close proximity with the vesicular export machinery.

Another key question surrounds the apparent loss of coupling to Gαi following recruitment to the immunological synapse (Molon et al., 2005). We report that the predominant Gαi protein in T cells, Gαi2, is actively excluded from most of the synapse rapidly upon its formation and remains partially depleted throughout its lifetime (Figure 9B). Given the active recruitment of CXCR4 and other GPCRs into the synapse, this could be one contributing reason for the loss of Gαi-coupling, however several other factors could also influence this [e.g., Src kinase-mediated phosphorylation of the DRY motif (Hauser et al., 2016)]. We were unable to achieve reasonable expression of tagged Gαq and so cannot compare the distribution of this or other G proteins with Gαi2. The nature of Gαi2 exclusion is also unclear, although we observe that is dependent on formation of an active synapse with engagement of adhesion molecules, and on both actin and microtubule integrity. G proteins interact with microtubules (Schappi et al., 2014), although Gαi2 distribution consistent with microtubule interactions was not clearly evident in our TIRF-SIM experiments. There are several other possible mechanisms for Gαi2 depletion in the synapse—including localized depalmitoylation (Wedegaertner, 2012), lipid packing-induced segregation (Oh and Schnitzer, 2001), or association with actively excluded partners—however the present data do not provide insight into which may be correct.

The findings that CXCR4 appears to segregate from both TCR and Gαi2, and that costimulatory potential can be recovered by receptor mutants deficient in phosphorylation at the C terminus or DRY motif raise the question of how it might deliver co-stimulatory signals within the IS. We did not examine the relative organization of CXCR4 with other components of the T cell activation process, nor the overall features of the synapse in the presence/absence of costimulation through CXCR4. It is therefore possible that CXCR4 may colocalise with costimulatory receptors (e.g., CD28) or adaptor proteins (e.g., LAT) and increase their activity by corecruitment of Lck; or by affecting global organization of the synapse—e.g., increasing integrin accumulation. Both of these models would be consistent with our observations of greater CXCR4- (and GPCR-)dependence in naïve T cells, which are more reliant on both CD28 costimulation and stable synapse formation. CXCR4 is also known to interact with other GPCRs, such as CCR5 (Contento et al., 2008; Felce et al., 2019), and it is also possible that it is able to influence signaling from these receptors within in the IS. Regardless of mechanism, we observe a clear independence on ligation, and so it seems likely this can be regulated primarily by overall CXCR4 expression.

Through a knockout screen of the 28 GPCRs in primary CD4^+^ T cells we observed a significant contribution of several receptors upon characteristic responses to activation. In some cases, particularly S1PR1 and GPR183, the magnitude of these effects was unexpectedly large, especially in naïve cells. This is in keeping with the greater need for costimulation in these cells (Dubey et al., 1996), however it could also relate to differences in underlying receptor expression as this was not assessed. It could also be due to differences in their accompanying APCs (moDCs for naïve, B cells for blasts), however given that differences were also observed when both were activated use anti-CD3/CD28 beads this seems unlikely. T cell stimulation by APCs was markedly more sensitive to GPCR knockout than stimulation by beads, with several receptors reporting effects in the former but not the latter. This could be due to stronger activation by beads vs. APCs, thereby masking more subtle contributions from GPCRs. Alternatively, the presence of APC-derived factors, both secreted ligands and cell-surface proteins, may be required for costimulation in some cases. Knockout of CD97, e.g., significantly impacted activation of naïve T cells by moDCs but not by beads, which may be due to its capacity to bind integrins (Wang et al., 2005) not present on beads. For GPCRs exhibiting effects on both APC- and bead-mediated stimulation, the presence of APC-derived ligands seems unlikely to have fully contributed to the observed effects. However, the presence of T cell-endogenous ligands cannot be discounted—particularly in the case of the sphingosine-1-phosphate and lysophosphatidic acid receptors, which are believed to engage their lipid ligands directly from the local membrane (Hanson et al., 2012). Nonetheless, even if endogenous ligation of such GPCRs is required for effects on T cell activation, this can be considered the baseline state of these receptors in these cells, and hence they would still possess intrinsic influence on cellular responses. This may be reflected in the large proportion of identified receptors that recognize lipid or lipid-soluble ligands (LPAR2, LPAR6, GPR174, GPR183, PTGER2, PTGER4, S1PR1, S1PR4), however this is broadly overshadowed by the stronger correlation with expression level. In general, those receptors identified as influencing T cell activation have a range of other known functions. Several classically mediate cell migration—e.g., chemokine (CXCR4, CCR7), sphingolipid (S1PR1, S1PR4), phospholipid (LPAR2, LPAR6), or oxysterol (GPR183) receptors – whereas others mediate sensitivity to proinflammatory (e.g., PTGER2, PTGER4) and/or immunoregulatory (e.g., P2RY8) ligands. All are members of the *Rhodopsin*-family of GPCRs with the sole exception of the *Adhesion*-family receptor CD97, and a large majority are known to couple preferentially to Gαi proteins.

Interpretation of these data must include a number of considerations. Firstly, knockout of each gene was targeted using a single guide RNA, and although each was selected for minimal off-target effects (Supplementary Table 2) we cannot fully exclude the possibility of contributions from other affected genes. Secondly, CD28 was engaged in all experiments and so the observed effects could arise from influences on either proximal or downstream signaling from TCR, CD28, or both. CD28 was not engaged when cells were stimulated with BSLBs, which may explain the lack of reported knockout effects on protein transfer. Alternatively, since the effects of GPCR knockout were typically more significant in naïve than blasted T cells, it is possible that ligand-independent GPCR costimulation disproportionately influences T cell activation over effector function. Furthermore, we do not dispute the possibility for ligand-dependent contributions of GPCRs not examined here (described for adenosine (Linnemann et al., 2009), and adrenaline (Fan and Wang, 2009) receptors, among others). Nonetheless, given the overall correlation between transcript abundance and knockout effect, it seems unlikely that GPCRs with very low expression contribute strongly in a ligand-independent manner.

Despite substantial differences in the effects of knockout across different GPCRs, we observed no evident correlation between receptor distribution or dynamics and costimulatory function. All studied receptors exhibited consistent centripetal migration yet no correlation with TCR. There were differences in the extent of central accumulation, however this likely stems from differences in the underlying rate of internal trafficking either to or from the synapse. These commonalities may hint at a possible shared mechanism for GPCR redistribution within the synapse, with emergent effects on T cell costimulation depending heavily on receptor-specific properties.

Our observations offer new insights into the contributions of GPCRs to T cell activation, and the nature of their organization within the T cell immunological synapse. Nonetheless, many outstanding questions remain, including how active receptor redistribution relates to costimulatory effects; how this is affected by the local distribution of G proteins; and why it appears to be largely disconnected from receptor ligation, at least in the case of CXCR4.

## Materials and Methods

### Primary CD4^+^ T Cell Isolation

Primary human CD4^+^ T cells were isolated using the RosetteSep Human CD4^+^ T Cell Enrichment Cocktail (StemCell Technologies) as per the manufacturer's instructions from leukocyte cones provided by UK National Health Service Blood and Transplant. Isolated cells were cultured in RPMI-1640 supplemented with 10% FCS, 4 mM L-glutamine, 10 mM HEPES, 1% non-essential amino acid solution (Gibco), and 1% penicillin-streptomycin solution (Gibco) at 37°C, 5% CO_2_. T cell blasts were generated by stimulating cells between 24 and 72 h after isolation. Cells were diluted to 1 × 10^6^/ml in supplemented RPMI-1640 containing 50 U/ml recombinant IL2 (PeproTech) and anti-human CD3/CD28 Dynabeads (Gibco) at 1 × 10^6^/ml. Cells were cultured for 3 days then beads were removed by magnetic separation and the medium replaced with fresh supplemented RPMI-1640 + 50 U/ml IL2. Cells were cultured for a further 4 days with medium replaced and cells diluted to 1 × 10^6^/ml as required.

HA-restricted clone 40 cells were generated as described previously (Peng et al., 2015). Briefly, peptide-specific T cells were isolated using IFNγ secretion assay and cloned by limiting dilution. Single cells were cultured with feeder cells (irradiated, pooled PBMCs from 2 to 3 healthy donors at a total cell concentration of 2 × 10^6^ cells/ml in RPMI 1640 supplemented with 10% heat-inactivated AB human serum and 30 μg/ml of PHA). IL2 was added on day 3 and replaced every 2–3 days. Every 14–16 days, T cell clones were restimulated with feeder cells as mentioned above. Antigen specificity of the T cell clone was assessed with intracellular cytokine staining after each round of expansion.

### pGEM Vector Cloning and mRNA Preparation

mRNA for transfection of exogenous proteins was produced *in vitro* from the T7 promoter-containing pGEM vector using the mMESSAGE mMACHINE T7 Transcription Kit (Thermo Fisher Scientific) as per the manufacturer's instructions. Genes encoding proteins of interest were directly synthesized as gene strings using the GeneArt service (ThermoFisher) and ligated into pGEM following digestion with *AgeI* and *HindIII*. For HaloTag-fused constructs these were followed by a short sequence encoding a GSGSG flexible linker and then the *HaloTag* gene at the 3′ terminus. For *GNAi2-SNAP-tag*, the *SNAP-tag* gene was inserted between nucleotides 342 and 343, corresponding to residues A114 and E115 in the αB-αC loop of Gαi2, following a short GSG linker. This tagging site has been demonstrated previously to retain Gαi2 activity (van Unen et al., 2016).

### mRNA Transfection

Cells were transfected with *in vitro*-prepared mRNA 24 h before imaging. Cells were washed three times with OptiMEM (Gibco) at room temperature and resuspended at 2.5 × 10^6^ cells/100 μl. 2.5−10 μg of the appropriate mRNA stock was added to 2.5 × 10^6^ cells, which were gently mixed, transferred to a Gene Pulser cuvette (BioRad) and pulsed for 2 ms at 300 V in an ECM 830 Square Wave Electroporation System (BTX). Cells were then immediately transferred to supplemented RPMI-1640 at 1 × 10^6^/ml and cultured for 24 h. The amount of mRNA used was optimized for each T cell donor and mRNA preparation by performing multiple transfections with titrated mRNA amounts.

### Cas9-Dependent Tagging of Endogenous CXCR4

Endogenous CXCR4 in Jurkat E6.1 cells was genetically fused to HaloTag at the C-terminus through Cas9-targetted homology-directed repair. The pSpCas9(BB)-2A-Puro (pX459) v2.0 vector (Ran et al., 2013) was obtained as a gift from Feng Zhang (Addgene plasmid #62988), into which the sequence 5′-TCTTTTACATCTGTGTTAGC-3′ was inserted to target Cas9 to the 3′ end of the *CXCR4* gene. Homology templates were generated by sequential nested PCRs to generate a fragment consisting of the 1 kb upstream and 1 kb downstream of the genomic cut-site flanking the *HaloTag* gene containing a terminal STOP codon. This was blunt-end ligated into the pJET1.2 shuttle vector (ThermoFisher). Nine μg of pJET1.2 HDR template and 1 μg pX459 were transfected into 2x10^6^ Jurkat E6.1 cells using the 100 μl Neon Transfection System (ThermoFisher) with settings: 1,325 V, 10 ms, three pulses. Cells were transferred to supplemented RPMI-1640 and cultured in the presence of 10 μM SPE7 pyrazine (a NHEJ inhibitor; Sigma-Aldrich) and 10 μM RS-1 (an HDR promoter; Sigma-Aldrich) for 7 days. Cells were stained with JanliaFluor 646 HaloTag ligand (Promega; see “HaloTag and SNAP-tag labeling”) and the HaloTag^+^ population sorted using a FACSAria III cell sorter (BD Biosciences). Correct tagging was confirmed by correlative TIRFM in both HaloTag and anti-CXCR4 channels.

### HaloTag and SNAP-Tag Labeling

HaloTag- and SNAP-tag-fused constructs were labeled through incubation with their requisite fluorescent ligand (200 nM JaneliaFluor 646 HaloTag ligand (Promega), or 500 nM SNAP-Cell 647-SiR ligand (New England BioLabs), respectively) in supplemented RPMI-1640 for 30 min at 37°C, washed three times, incubated for a further 30 min then washed once and used immediately for imaging.

### SLB Preparation and Use

SLBs were prepared as described previously (Choudhuri et al., 2014). Briefly, micelles of 1,2-dioleoyl-sn-glycero-3-phosphocholine (Avanti Polar Lipids Inc.) supplemented with 12.5% 1,2-dioleoyl-sn-glycero-3-[(N-(5-amino-1-carboxypentyl) iminodiacetic acid) succinyl]-Ni (Avanti Polar Lipids Inc.) were flowed onto glass coverslips hydroxylated with piranha solution, plasma cleaned, and affixed with adhesive 6-lane chambers (Ibidi). SLBs were blocked and washed, then incubated with recombinant His-tagged proteins of interest (all produced in-house except HLA-DRB1^*^09:01-HA, which was obtained from the NIH tetramer facility) at the requisite concentrations to achieve the desired density: 30 molecules/μm^2^ for UCHT1-Fab and HLA-DRB1^*^09:01-HA, 200 molecules/μm^2^ for ICAM1. The specific combination of unconjugated proteins or proteins conjugated to different dyes (AlexaFluors 405, 488, 568, and 657) was varied to suit the demands of each experiment. Within 2 h of preparation, SLBs were pre-warmed to 37°C and cells were infused into the SLB chambers at ~5 × 10^5^/lane. Samples were either imaged live or fixed with warm 4% para-formaldehyde in PBS. During experiments in which soluble CXCL12 was present, recombinant CXCL12 (PeproTech) was added to a final concentration of 0.1 μg/ml in the imaging buffer prior to cell exposure to SLB. In order to present CXCL12 on SLB, 0.005% biotinylated 1,2-dioleoyl-sn-glycero-3-phosphoethanolamine (Avanti Polar Lipids Inc.) was included in the SLB preparation then loaded with 4 μg/ml streptavidin for 20 min. After washing, CXCL12-biotin (Chemotactics) was then added at 100 ng/ml for 20 min to allow capture by the SLB-presented streptavidin at a density of 100 molecules/μm^2^.

### GUV Preparation and Use

GUVs were prepared using an electro-formation method. One mg/ml lipid mixture (POPC:nickelated lipid, 96:4 molar ratio) was deposited on platinum wire, dried, and dipped into a Teflon-coated chamber filled with 300 mM sucrose. GUV formation was triggered by a 10 Hz AC field for 1 h which was followed by 2 Hz for 30 min. After formation, 100 μL of the GUV suspension was incubated with 1 μg/ml His-tagged protein for 30 min.

UCHT1/ICAM-bearing GUVs were mixed with Gαi2-SNAP-tag-expressing CD4^+^ T cells in L-15 medium (Sigma-Aldrich) containing 0.1 μg/ml anti-CD45 Fab fragment (Gap8.3 clone) conjugated to AlexaFluor 647. Live cell-GUV contacts were imaged by confocal microscopy after 10–30 min incubation at 37°C. In conditions using selective inhibitors, the relevant compound was added 15 min after cell-GUV mixing, and contacts imaged 15 min later. These were nocodazole (10 μg/ml final concentration; Sigma-Aldrich), latrunculin-A (1 μg/ml final concentration; Sigma-Aldrich), or PP2 (10 μM final concentration; Sigma-Aldrich). The exception was PTx (Tocris Bioscience), which was added to the cells in normal culture medium 18 h before imaging to a final concentration of 2 μg/ml.

### Glass Coating for Cell Activation

For activation experiments on glass without SLB, 8-well μ-slide chambers (Ibidi) were coated with either PLL or anti-CD3/CD28 prior to cell loading. PLL was applied by incubation of 250 μl/well 0.01% PLL (Sigma Aldrich) in dH_2_O for 15 min followed by 3 washes with 300 μl PBS. For antibody coating, wells were first coated with 250 μl 50 μg/ml polyclonal donkey anti-mouse antibody (ThermoFisher Scientific) in coating buffer (50 mM Na2CO3, 50 mM NaHCO3, pH 9.6, filtered using a 0.22 μm Millex®-GP syringe filter unit) at 4°C overnight, then washed with 3 x 300 μl PBS and incubated with 250 μl mouse anti-CD3 (OKT3; BioLegend) and mouse anti-CD28 (CD28.2; eBioscience) at 5 μg/ml in PBS for 1 h before final 3 × 300 μl PBS washes.

### Microfluidic Chamber Preparation and Use

For the formation and imaging of the T cells conjugates we followed the approach detailed in Jang et al. (2015). The device design is the same as previously described but the fabrication technique differs slightly. The device comprised two parts, top and bottom, that were fabricated separately and assembled before use. The top and bottom masters were made using SU8 2015 photoresist (MicroChem) with a height of 30 and 15 μm, respectively. Polydimethylsiloxane (PDMS) soft lithography (SYLGARD®184 kit, Dow Corning) was used to fabricate the microfluidic device with base to curing agent ratio 10:1. For the bottom part a thin layer of PDMS (approximately 100 μm) was spun on the master and on a glass microscopy coverslip (Menzel Gläser) which was then carefully positioned on top of the device before curing on a hot plate at 70°C for 40 min. For the top part the curing was done in an oven at 80°C for 1 h. The two parts were then plasma cleaned and assembled under an inverted microscope with the aid of a drop of methanol to ease positioning. After assembly the device was put under vacuum for bonding. Prior to use, devices were filled with PBS + 5% BSA and left to block overnight, before washing and refilling with supplemented RPMI-1640, taking care to avoid the introduction of bubbles.

For conjugation experiments, 1 × 10^7^ Raji B cells were incubated in 10 ml supplemented RPMI-1640 containing SEE (Toxin Technology) at 1 μg/ml and CellTracker Green CMFDA (ThermoFisher Scientific) at 10 μM for 30 min at 37°C, pelleted at 300 × g for 5 min and washed with 10 ml fresh medium, repeating three times. For experiments with monensin treatment, 7 μl GolgiStop Protein Transport Inhibitor solution (BD Biosciences) was also added to the cells 6 h before SEE incubation. Cells were then resuspended in RPMI-1640 at 1 × 10^7^/ml, filtered with a 70 μm cell strainer (Fisher Scientific) and injected into the microfluidic device using a Legato 100 single syringe pump (WPI) at 5 μl/min for 5–10 min until most chambers were occupied with cells as observed down a white-light microscope. The device was removed from the pump and centrifuged in a swing-bucket centrifuge at 300 x g for 1 min. Jurkat E6.1 cells expressing CXCR4-HaloTag and pre-stained with JaneliaFluor 646 HaloTag ligand were introduced into the device at 1 × 10^7^/ml, 5 μl/min for 10 min, followed by 37°C RPMI-1640 at 5 μl/min for 20 min, during which the device was housed within an incubator at 37°C. Cells were then fixed with PBS + 4% PFA flowed in at 10 μl/min for 10 min, then washed with PBS at 10 μl/min for 20 min.

### TIRF, TIRF-SIM, and Confocal Microscopy

Conventional TIRFM was performed on an Olympus cellTIRF-4Line system using a 150× (NA 1.45) oil objective. Confocal images were acquired using a Zeiss 780 LSM using a 40× water objective (NA 1.2). Imaging of live samples was performed at 37°C, and of fixed samples at room temperature. Super-resolution imaging was performed on a custom-built TIRF-SIM setup based on a ferroelectric spatial light modulator used to generate diffraction patterns and adjust the TIRF angle (Li et al., 2015). The TIRF angle was selected to ensure below 150 nm penetration depth 488, 560, and 640 nm laser lines. Illumination and detection was performed through an Olympus 100× (NA 1.49; UPLAPO100XOHR) oil objective. Raw images were obtained on two Hamamatsu Orca Flash 4.0 cameras, and reconstructed with custom made software (Li et al., 2015). Multi-channel TIRF-SIM images were corrected for chromatic aberrations using the MultiStackReg plugin for ImageJ and 0.1 μm TetraSpeck microspheres (ThermoFisher Scientific) on glass as a reference standard.

### Image Analysis and Visualization

All image analysis and visualization was performed using the ImageJ software. Pearson correlation coefficients (PCCs) were calculated using the Coloc 2 plugin to perform pixel intensity correlation between channels. Only above-threshold pixels in either channel were included in the analysis to avoid false positive correlations. Radial averages were generated by using the transform function to rotate the starting image by all angles 1°-359°, then compressing the resultant transformations into a single stack and performing a z-projection of mean intensity. Radial averages from multiple cells were combined and averaged using the z-projection function following intensity normalization.

Three-dimensional z-stacks were visualized using the 3D-projection and orthogonal view functions. Comparisons of basal vs. distal intensity were performed by defining an area of 3 × 3 μm at x-y coordinates corresponding to the center of the synapse in the basal plane, then deriving the mean pixel intensity value within this area across all z positions. The peak intensity at the lower z position was taken as the basal intensity, and that at the higher z position as the distal intensity.

Analysis of intensity inside vs. outside GUV-cell contacts was performed using the multipoint tool function. Using the CD45 and UCHT1 signals to define the plasma membrane of the T cell and the site of the contact, the gray value intensity of individual pixels was measured at regular intervals of 0.5 μm within the contact and either side of the contact to a distance equivalent to 1× the width of the contact. The final intensity values inside and outside the contact were determined as the mean intensity across all measured pixels within that area.

### Single-Particle Tracking

Videos used for single-particle tracking were captured at 50 ms/frame for 15 s using TIRFM. Single-particle tracking analysis was performed in ImageJ using the TrackMate plugin (Tinevez et al., 2017), version 3.8.0. Spots were identified through sub-pixel localization using a difference of Gaussians filter with an estimated spot diameter of 0.5 μm, then filtered by quality. Frame-to-frame spot linking was performed using a Linear Assignment Problem tracker with a with a maximum linking distance of 1 μm, a maximum gap-closing distance of 1 μm, and a maximum gap-closing frame gap of one frame. Trajectory coordinates were characterized using the TraJClassifier plugin (Wagner et al., 2017), with a minimum track length of 30 frames, window size of 30 frames, minimum segment length of 30 frames, and resample rate of one. As a result, only tracks of at least 30 frames (1.5 s) were taken forward for characterization. Total numbers of cells imaged and tracks recorded are given in Supplementary Table 1. For trajectory analysis in which absolute position was important (i.e., track movement relative to defined cell regions or to synapse center), track x and y coordinates at each time point were compared to coordinate maps of each cell derived from single-frame images of region-defining channels (UCHT1, ICAM1, IRM) taken immediately prior to particle tracking, thereby sorting each frame of each track into one of the defined c, p, or dSMAC regions. Visualization of track positions were generated using GraphPad Prism 8, or the SankeyMATIC software (https://github.com/nowthis/sankeymatic).

### Primary B Cell and Monocyte Isolation, Differentiation, and Stimulation

Primary human B cells and monocytes were isolated using the RosetteSep Human B Cell and monocyte Enrichment Cocktails (StemCell Technologies) as per the manufacturer's instructions from leukocyte cones provided by UK National Health Service Blood and Transplant. Isolated cells were cultured in RPMI-1640 supplemented with 10% FCS, 4 mM L-glutamine, 10 mM HEPES, 1% non-essential amino acid solution (Gibco), and 1% penicillin-streptomycin solution (Gibco) at 37°C, 5% CO_2_. B cells were also cultured in the presence of 1 mM sodium pyruvate (Gibco), 50 ng/ml IL4 (PeproTech), 25 ng/ml IL2 (PeproTech), 100 ng/ml BAFF (BioLegend), and 100 ng/ml IL21 (BioLegend). Monocytes were differentiated into moDCs by culturing with 50 ng/ml IL4 (PeproTech) and 100 ng/ml GM-CSF (Immunotools) at 1 × 10^6^/cm^2^ in adherent culture for 6 days. Twenty-four h before use in T cell stimulation assays, moDCs were activated by addition of 1 μM prostaglandin E2 (Sigma-Aldrich), 50 ng/ml TNFα (PeproTech), 10 ng/ml IL1β (Bio-Techne), and 20 ng/ml IFNγ (Bio-Techne). Differentiation was confirmed by assessing expression of CD11c and CD86 (see “Flow cytometry”).

### Cas9 RNP Preparation and Transfection

Gene disruption in primary CD4^+^ T cells was performed by transfection with *in vitro*-prepared Cas9 ribonucleoprotein (RNP) complexes. For all targets, gene-specific Alt-RCRISPR-Cas9 gRNA was obtained from IDT (sequences given in Supplementary Table 2). To generate RNP complexes, 150 pmol Alt-RCRISPR-Cas9 gRNA was incubated with 150 pmol Alt-R tracrRNA (IDT) in nuclease-free duplex buffer (IDT) at 95°C for 5 min and resultant duplex allowed to cool to room temperature. One hundred and fifty pmol of Alt-R S.p Cas9 Nuclease V3 (IDT) and duplexed gRNA were mixed in 8 μl nuclease-free duplex buffer and incubated at 37°C for 15 min. One hundred and fifty pmol Alt-RCas9 Electroporation Enhancer (IDT) was added to the RNP solution, and the whole mix then added to 1.5 × 10^6^ naïve primary CD4^+^ T cells, which had previously been washed with room-temperature OptiMEM three times and resuspended in 50 μl OptiMEM. The cell-RNP mix was transferred to a Gene Pulser cuvette (BioRad) and pulsed for 2 ms at 300 V in an ECM 830 Square Wave Electroporation System (BTX). Cells were then immediately transferred to 500 μl supplemented RPMI-1640. Hundred μl of cells were removed and blasted for 3 days as described above, while the remaining ~1.2 × 10^6^ cells were left in resting culture until used in T cell stimulation assays.

### TIDE Analysis

The efficiency of gene disruption was determined using TIDE analysis (Brinkman et al., 2014). Genomic DNA (gDNA) was isolated from 0.5 × 10^6^ transfected CD4^+^ T cell blasts 7 days after RNP transfection using the GenElute Mammalian Genomic DNA Miniprep Kit (Sigma-Aldrich) as per the manufacturer's instructions. Isolated gDNA was then used as the template in polymerase chain reactions using the relevant oligonucleotide primers given in Supplementary Table 2, to amplify the ~500 bp region surrounding the predicted genomic cut site for each target. These products were sequenced using reversible terminator sequencing and the resulting chromatograms compared to those derived from PCR products of untransfected cell gDNA using the TIDE algorithm (Desktop Genetics). TIDE analysis outputs are given in Supplementary Figure 4.

### T Cell Stimulation Assay

Stimulation of knockout cells was performed 7 days post-transfection with RNP complexes. Both naïve and blasted CD4^+^ T cells were activated with anti-human CD3/CD28 Dynabeads (Gibco) or SEE-loaded antigen-presenting cells (APCs). moDCs were the APCs used for naïve T cells, B cells for blasted T cells. In each case, APCs and T cells were obtained from the same blood donor. Twenty four h before stimulation, T cell blasts were transferred to IL2-free growth medium and B cells were transferred to cytokine-free growth medium. Immediately before stimulation, T cells were centrifuged at 300 × g for 5 min and resuspended in supplemented RPMI-1640 at a density of 5 × 10^4^ / 50 μl. APCs were loaded with SEE (Toxin Technology) for 1 h at 37°C at concentrations ranging from 10^−4^ ng/ml to 10^3^ ng/ml or with no SEE, then washed four times with growth medium and resuspended at 1 × 10^5^ / 50 μl. Fifty μl of T cell suspension was added to either 50 μl APC suspension, 50 μl growth medium containing 1 × 10^5^ anti-human CD3/CD28 Dynabeads, or 50 μl growth medium alone in a U-bottomed 96-well plate, which was gently centrifuged at 25 × g for 1 min then returned to culture. After 2 h, 50 μl of growth medium containing 0.1 μl GolgiStop Protein Transport Inhibitor solution (BD Biosciences) was added to cells. After a further 4 h, cells were centrifuged at 300 × g for 5 min then fixed with 4% para-formaldehyde in PBS for 10 min before staining for flow cytometry.

### Flow Cytometry and Cell Sorting

Following T cell stimulation assays, samples were permeabilised with 0.1% saponin in PBS for 15 min, quenched with 100 mM glycine in PBS for 20 min, then blocked with 6% bovine serum albumin (BSA) in PBS for 2 h, with 3 PBS washes between each step. Following blocking, cells were incubated for 2 h with 1 μg/ml anti-CD69 (FN50; BioLegend), anti-IL2 (MQ1-17H12; BioLegend), anti-IFNγ (4S.B3; BioLegend), and either anti-CD19 (4G7; BioLegend) in the case of B-T cell conjugates, or anti-CD11c (3.9; BioLegend) for moDC-T cell conjugates, all in PBS + 3% BSA + 0.02% saponin. Cells were washed 3 times with PBS + 0.1% saponin and resuspended in 100 μl PBS + 1 mM EDTA for analysis. Samples were analyzed using the high-throughput 96-well plate sampler of a FACSCanto II Flow Cytometer (BD Biosciences). Data were analyzed using FlowJo version 8.8.7. T cells were gated first by FS/SS and then as the CD19/CD11c^−ve^ population (in the case of APC-T cell conjugates) or as the PE-Cy5^−ve^ PE-Cy7^−ve^ population (for bead-T cell conjugates). Thresholds at which cells were defined as positive for CD69, IL2, and IFNγ were determined by reference to cells stained with appropriate isotype controls. Response to stimulation was expressed as normalized ΔCD69/IL2/IFNγ^+^ cells, which was defined as the difference between the frequency of positive cells in a sample and that in the control sample consisting of T cells + APC with no SEE (for APC-T cell conjugates) or of T cells alone (for bead-T cell conjugates). This was then normalized to the maximum value observed for the CD19^−ve^ control, which was set at 100.

Alongside this, the unstimulated cell condition was stained with anti-TCR (IP26; BioLegend) and anti-CD28 (CD28.2; BioLegend) without prior permeabilisation at 1 μg/ml for 45 min then washed and analyzed in the same manner. For other experiments where surface staining was sufficient, cells were fixed and stained in the same manner as above, using the relevant antibodies in each case; one or more of anti-CXCR4 (12G5; BioLegend). anti-TCR (IP26; BioLegend), or anti-CD28 (CD28.2; BioLegend).

A pure CXCR4^−ve^ population was obtained for mutant CXCR4-HaloTag transfection by fluorescence-activated cell sorting. Cells were stained with 1 μg/ml anti-CXCR4 (12G5; BioLegend) in PBS + 2% FCS on ice for 30 min, then washed 3 times with cold PBS + 2% FCS and the negative population sorted using a FACSAria III cell sorter (BD Biosciences).

### Bead Supported Lipid Bilayers (BSLB)

Unfunctionalised silica beads (5.0 μm diameter, Bangs Laboratories, Inc.) were washed extensively with PBS in 1.5 ml conical microcentrifuge tubes. BSLBs were formed by incubation with mixtures of liposomes to generate a final lipid composition of 0.2 mol% Atto-DOPE Atto565; 12.5 mol% DOGS-NTA in DOPC at a total lipid concentration of 0.4 mM. The resultant BSLBs were washed with 1% human serum albumin (HSA)-supplemented HEPES-buffered saline (HBS), subsequently referred to as HBS/HSA. To saturate NTA sites, BLSBs were then blocked with 5% casein 100 μM NiSO_4_ for 20 min. After two washes, BSLBs were loaded with concentrations of His-tagged proteins required to achieve the indicated molecular densities (see figure legends). Excess proteins were removed by washing with HBS/HSA after 30 min. Primary CD4^+^ T cell blasts (2.5 × 10^5^/well) were incubated with BSLBs at 1:1 ratio in a V-bottomed 96 well plate (Corning) for 90 min at 37°C in 100 μl HBS/HSA. For gentle dissociation of BSLB-cell conjugates, culture plates were gradually cooled down by incubation at RT for 15 min, followed by incubation on ice. After 45 min, cells and BSLBs were pelleted at 300 x g for 5 min prior to resuspension in ice-cold 5% BSA in PBS pH 7.4. The single BSLBs and cells were gently resuspended prior to staining for flow cytometry analysis.

### Multicolour Flow Cytometry of BSLBs

Staining with fluorescent dye-conjugated antibodies was performed immediately after dissociation of cells and BSLB conjugates. Staining was performed in ice-cold 5% BSA in PBS pH 7.4 (0.22 μm-filtered) for a minimum of 30 min at 4°C with agitation to avoid BSLB sedimentation (700 rpm in the dark). Cells and BSLBs were then washed three times and acquired immediately using an LSRFortessa X-20 flow cytometer equipped with a high-throughput sampler. For absolute quantification, we used Quantum Molecules of Equivalent Soluble Fluorescent dye (MESF) beads (see below), which were first acquired to set photomultiplier voltages to position all the calibration peaks within an optimal arbitrary fluorescence units' dynamic range (between 10^1^ and 2 × 10^5^, and before compensation). Fluorescence spectral overlap compensation was then performed using unlabelled BSLBs and cells, and single color-labeled cells and BSLBs. For markers displaying low surface expression levels unstained and single color stained UltraComp eBeads (Thermo Fisher Scientific Inc.; #01-2222-42) were used for the calculation of compensation matrixes. Resulting compensation matrixes were applied and experimental specimens and Quantum MESF beads were acquired using the same instrument settings. In most experiments acquisition was set up such that a minimum of 5 × 10^4^ single BSLBs were recorded.

### Transwell Migration Assay

6.5 mm transwell inserts with 5 μm pore polycarbonate membranes (Scientific Laboratory Supplies) were coated overnight at 4°C with 100 μl PBS containing 50 μg/ml hICAM1 and either anti-CD3 (UCHT1) or mouse IgG1 isotype control at 50 μg/ml. Inserts were then washed 3 times and blocked with PBS + 5% BSA for 2 h at 4°C then washed three times with OptiMEM. Six hundred μl supplemented RPMI-1640 containing 0 or 0.25 μg/ml CXCL12 (PeproTech) was added to wells of a 24-well plate, on top of which the insert was carefully overlaid then filled with 1 × 105 T cells in 100 μl supplemented RPMI-1640. Cells were allowed to migrate for 1 h at 37°C then the total number of cells in the bottom chamber was counted using a FACSCanto II Flow Cytometer (BD Biosciences). Cell numbers were Normalized to wells in which 100 μl cell suspension was added directly to the bottom chamber.

### Statistical Analysis

All statistical tests were done with GraphPad Prism 8 software. The appropriate statistical test for each experiment is noted in the figures. The number of independent replicates in each case is provided in the associated figure legend.

## Data Availability Statement

The raw data supporting the conclusions of this article will be made available by the authors, without undue reservation.

## Author Contributions

JHF and MLD conceived and led the study, secured primary funding, and wrote the manuscript. JHF performed most experimental work and prepared the figures. LP designed and prepared the microfluidic devices, with practical support from MJ, and intellectual and financial support from DA and JF. ES performed the GUV experiments. PFC performed the BSLB transfer experiments. KK assisted with the TIRF-SIM experiments. MF and KK established and maintained the TIRF-SIM system, with MF and MLD securing funding. YP and TD generated and maintained the HA-restricted T cell clone. All authors provided helpful feedback on the preparation of the manuscript.

## Conflict of Interest

The authors declare that the research was conducted in the absence of any commercial or financial relationships that could be construed as a potential conflict of interest.

## Supplementary Material

Supplementary Figure 1 **(A)** TIRFM image examples of primary CD4^+^ T cell blasts expressing CXCR4-HaloTag interacting with UCHT1- and ICAM1-containing SLBs for 5, 10, and 30 min. **(B)** Max-intensity projections and orthogonal views of a confocal microscopy z-stack of CD4^+^ T cell blast expressing CXCR4-HaloTag on SLB presenting ICAM1 and UCHT1 for 30 min. **(C)** TIRFM image examples of primary CD4^+^ T cell blasts prestained with anti-CXCR4 mAb interacting with UCHT1- and ICAM1-containing SLBs for 30 min. **(D)** TIRFM image examples of HA-DRB1-specific CD4^+^ T cells expressing CXCR4-HaloTag and pre-stained with anti-TCR mAb interacting with SLB presenting ICAM1 and pHA-MHC for 10 and 30 min. **(E)** TIRFM images (left), cross-sectional normalized intensity profile (center), and radial averages (right) for CD4^+^ T cell blasts expressing CXCR4-HaloTag and stained for F-actin with phalloidin, on activating SLB for 10 min and treated with indicated inhibitors. Plots are mean normalized intensity at each position ± std dev. **(F)** Histogram of straightness for single particle CXCR4-HaloTag trajectories with different diffusive properties under activating and non-activating conditions. **(G)** Histogram of cell displacement over 20 min incubation for CD4^+^ T cell blasts on ICAM1-containing SLB in the presence of soluble and surface-presented CXCL12, measured by time-lapse IRM. **(H)** Histograms of mean diffusion speed and straightness for single particle CXCR4-HaloTag trajectories with different diffusive properties under activating and conditions in the presence of soluble and surface-presented CXCL12. All scale bars are 5 μm.

Supplementary Figure 2 **(A)** Example flow cytometry histogram of anti-CXCR4 staining on wt and CXCR4^−ve^ CD4^+^ T cell blasts transfected with HaloTag-fused CXCR4 mutants. **(B)** Example flow cytometry histogram of anti-CXCR4 staining on wt and CXCR4^−ve^ CD4^+^ T cell blasts transfected with untagged CXCR4 mutants. **(C)** Example flow cytometry histograms of anti-CD69, -IL2, and -IFNγ staining on wt and CXCR4^−ve^ CD4^+^ T cell blasts transfected with untagged CXCR4 mutants and stimulated with anti-CD3/CD28 beads for 6 h.

Supplementary Figure 3 **(A)** Representative TIRFM examples of Gαi2-SNAP-tag-expressing CD4^+^ T cell blasts interacting with or activating SLB for 10 or 30 min. **(B)** Max-intensity projections and orthogonal views of confocal microscopy z-stacks of CD4^+^ T cell blasts on glass coated with PLL or anti-CD3/CD28 antibodies. **(C)** TIRF-SIM examples of Gαi2-SNAP-tag in CD4^+^ T cell blasts on activating SLB for 10 min. All scale bars are 5 μm.

Supplementary Figure 4 **(A)** Example histograms of flow cytometry data of anti-CD69 staining on wt and CD3^−ve^ CD4^+^ T cells (blast and naïve) from the same donor following 6 h stimulation with anti-CD3/CD28 beads or APCs loaded with the indicated concentration of SEE. **(B)** Example flow cytometry data for CD3, CD28, or CXCR4 expression in wt CD4^+^ T cells (blast and naïve) and cells transfected with Cas9 RNP complexes targeting the relevant gene (“KO”). **(C)** Example sequencing chromatograms used for TIDE analysis. *CXCR3* sequences in wt and CXCR3 KO cells were compared to identify regions of aberrant signal (bottom), which then underwent decomposition to determine the relative frequencies of different indel mutations. **(D)** Pooled TIDE data from all Cas9 screen experiments. Values indicate the percentage of the sequencing data that corresponds to fully wild-type sequence. Boxes are mean with min-max; each symbol indicates a different donor. **(E)** Normalized CD3, CD28, and CD69 expression levels as measured by flow cytometry in unstimulated CD4^+^ T cell blasts following knockout of the indicated genes. Values are normalized to the gMFI value in wt cells. Boxes are mean with min-max; each symbol indicates a different donor. **(F)** Example flow cytometry histograms of anti-CD69, -IL2, and -IFNγ staining on unstimulated wt and individual-gene knockout CD4^+^ T cells (blast and naïve) from a single donor. **(G)** Absolute transfer of TCRαβ (left) or CD40L (right) from CD4^+^ blasts to BSLBs presenting ICAM1, CD40, and titrated densities of UCHT1. Plots show mean ± std dev., with best-fit non-linear response curves for each target. ^***^*p* < 0.001.

Supplementary Table 1 Summary of cell and track numbers for single-particle tracking experiments. All samples were examined in three independent experiments using cells derived from different donors.

Supplementary Table 2 Summary of Cas9 gRNA and TIDE oligonucleotide sequences used in knockout of all investigated genes. All sequences are given 5′-3′. On- and off-target scores are those provided by the gRNA supplier (IDT).

Supplementary Movie 1 Example of single-particle tracking of CXCR4-HaloTag in primary CD4^+^ T cells on SLB. Images are raw acquisition (left), spot detection (center), and frame-to-frame tracking (right).

Supplementary Movie 2 Example videos of CXCR4-HaloTag in primary CD4^+^ T cells on resting (left) and activating (center & right) SLB at the indicated time points.

Supplementary Movie 3 Example videos of mutant CXCR4-HaloTag in CXCR4^−ve^ CD4^+^ T cells on activating SLB 10 min activation.

Supplementary Movie 4 Example video of Gαi2-SNAP-tag in primary CD4^+^ T cells landing on activating SLB. UCHT1 is shown in green, ICAM1 in blue, Gαi2 in red, IRM in gray.

Supplementary Movie 5 Example videos of HaloTagged GPCRs in primary CD4^+^ T cells on activating SLB 10 min after activation.
