# Supplementary figures and images for: Single-Molecule, Super-Resolution, and Functional Analysis of G Protein-Coupled Receptor Behavior Within the T Cell Immunological Synapse
Source: Front Cell Dev Biol. 2021 Jan 18;8:608484. doi: 10.3389/fcell.2020.608484 (PMC7848080; doi:10.3389/fcell.2020.608484)

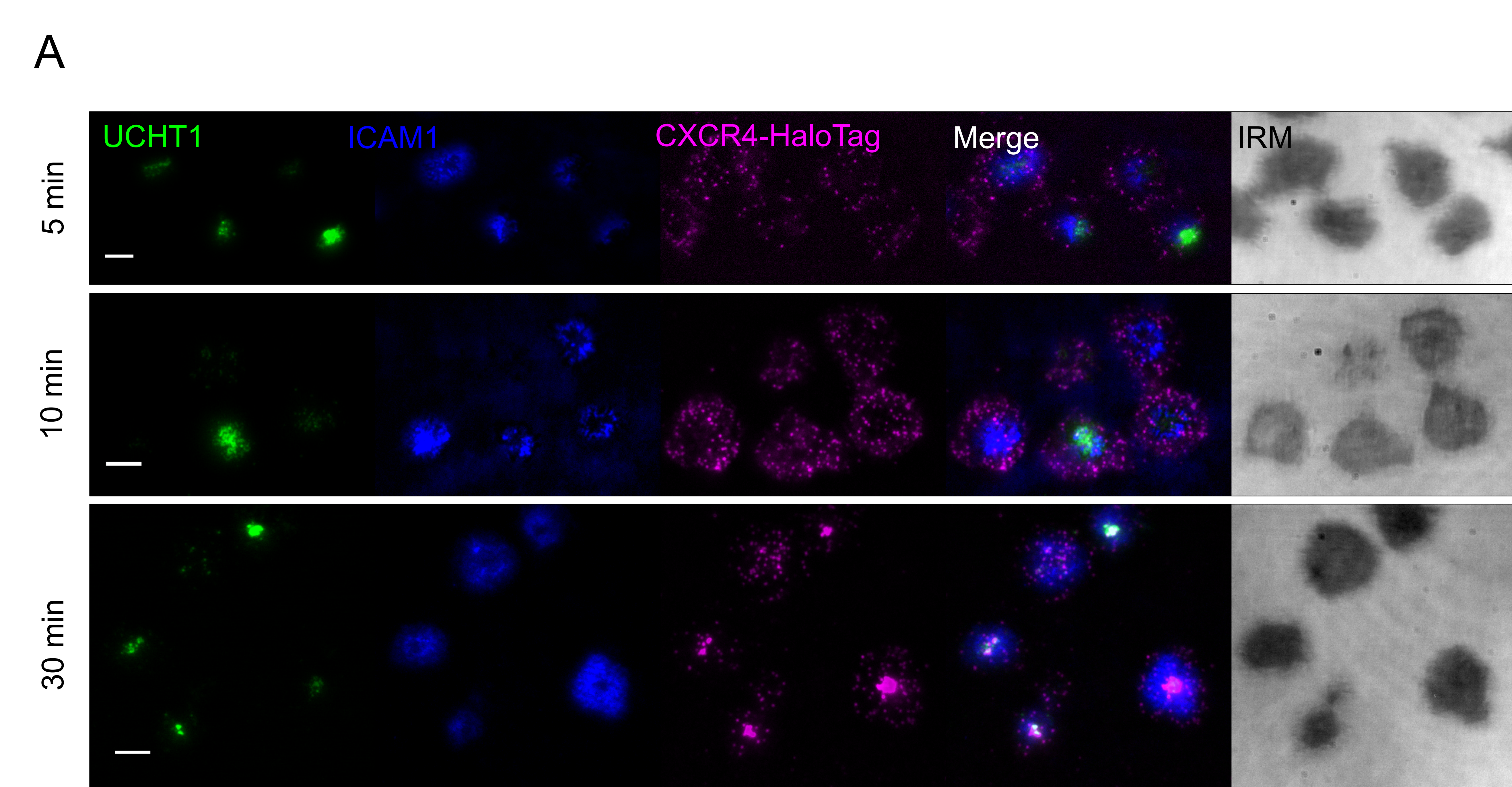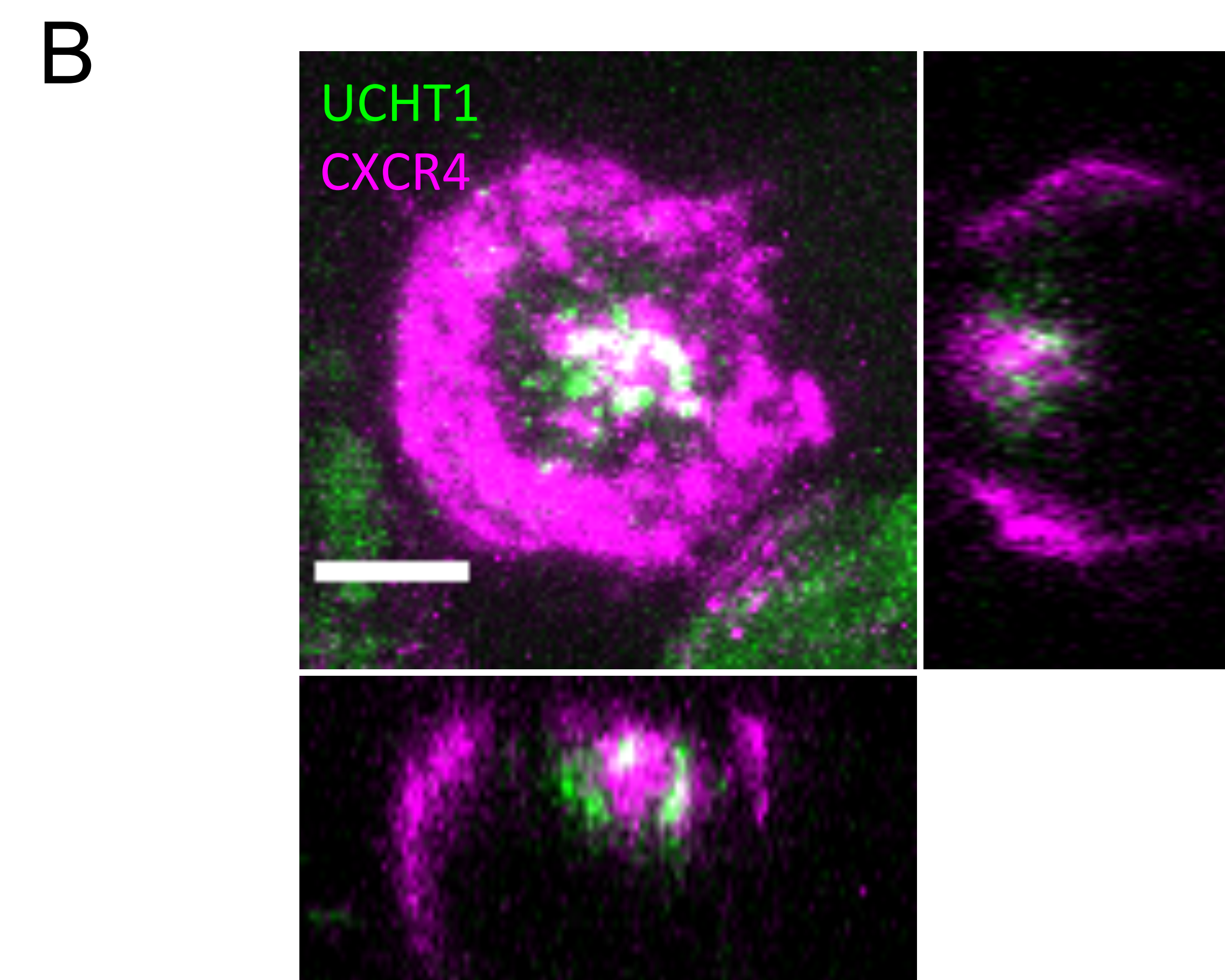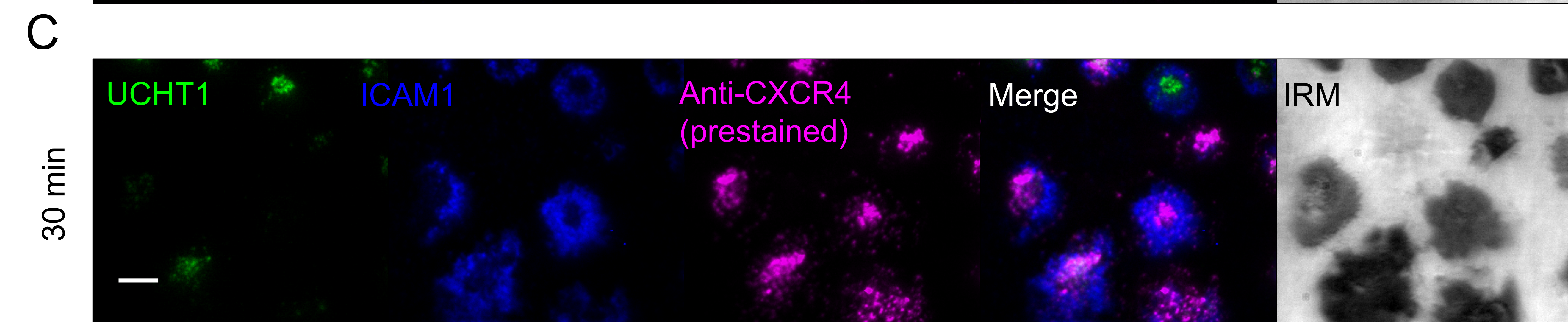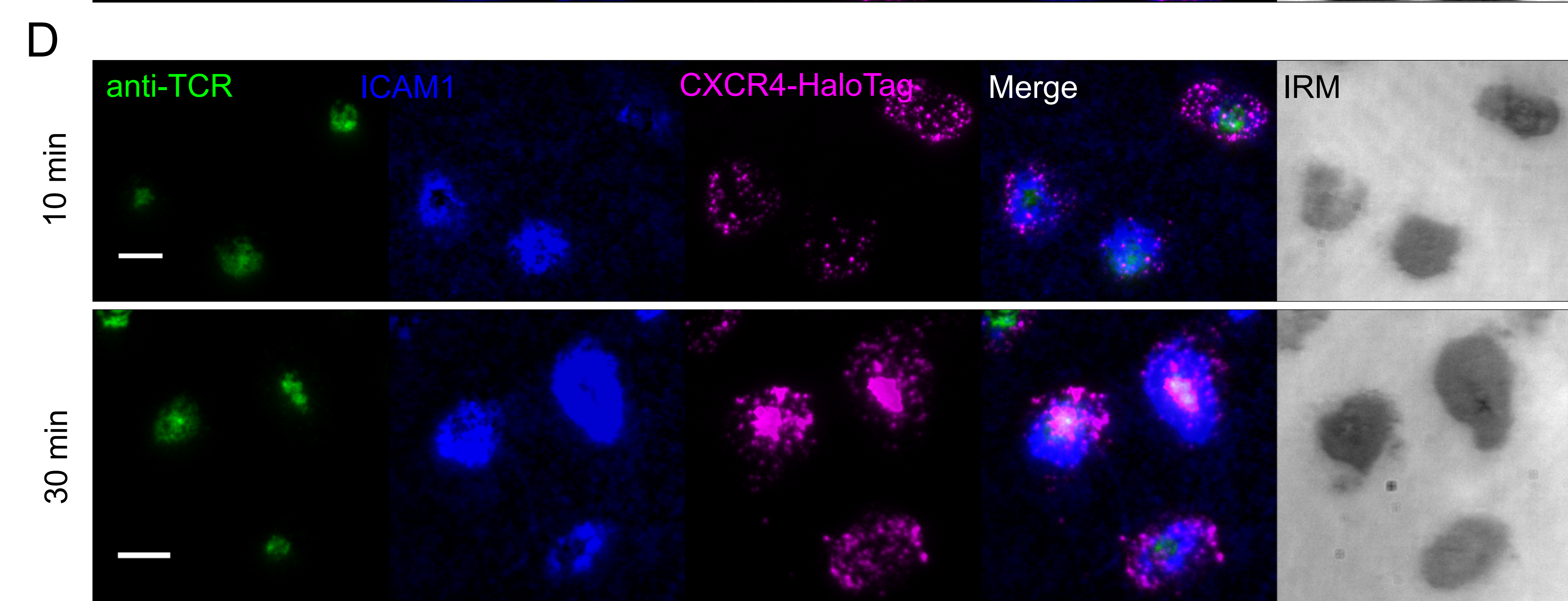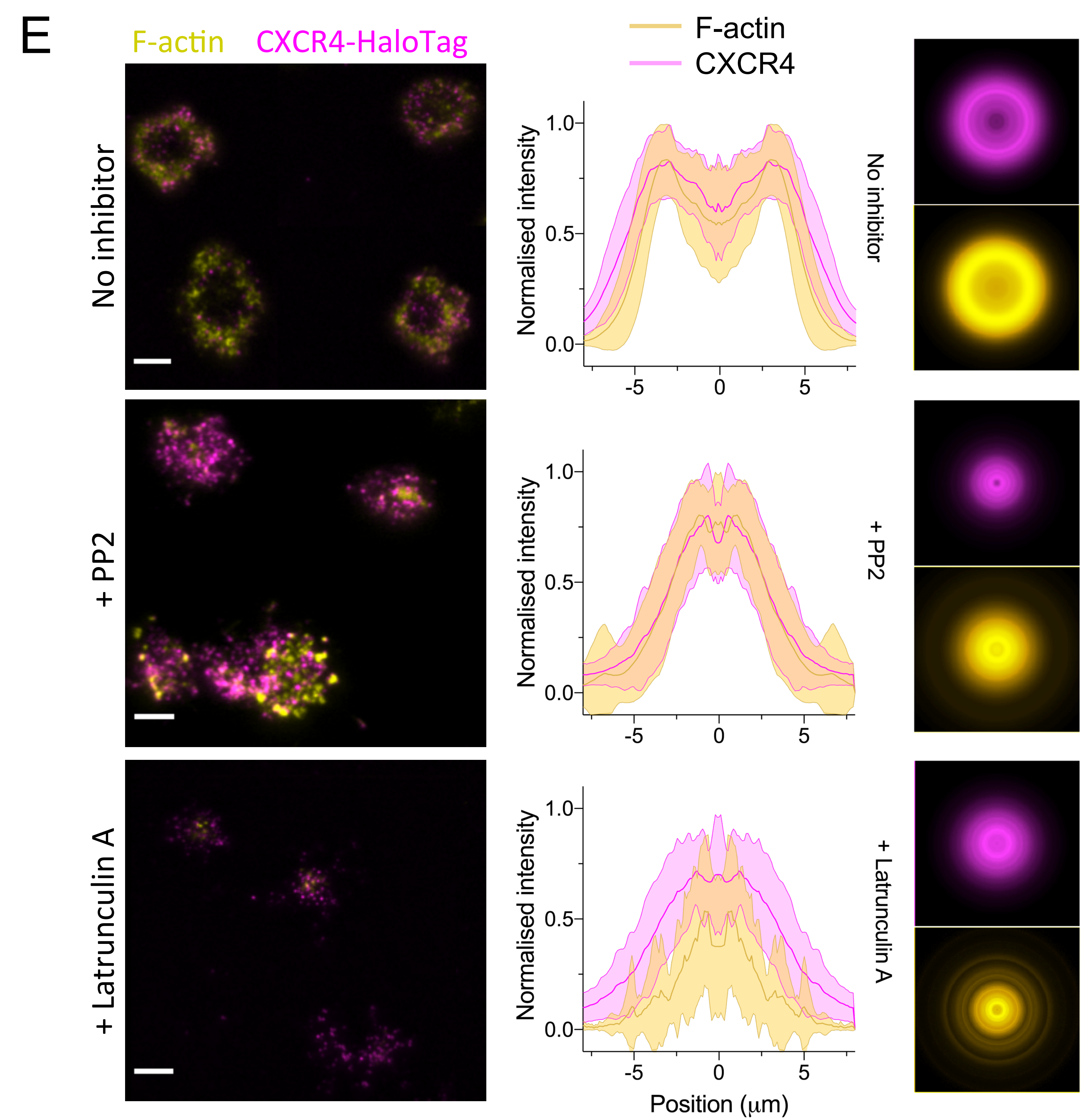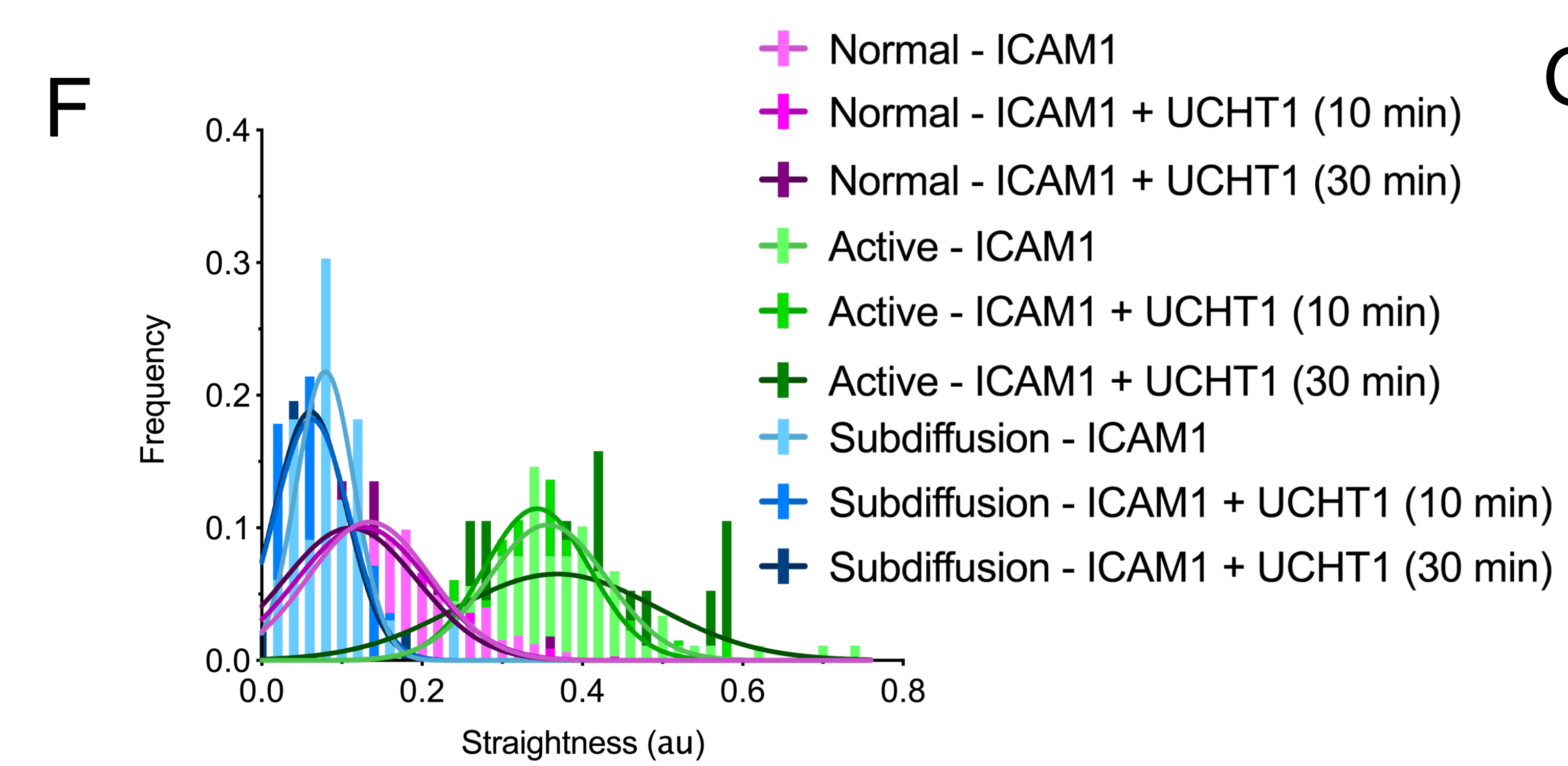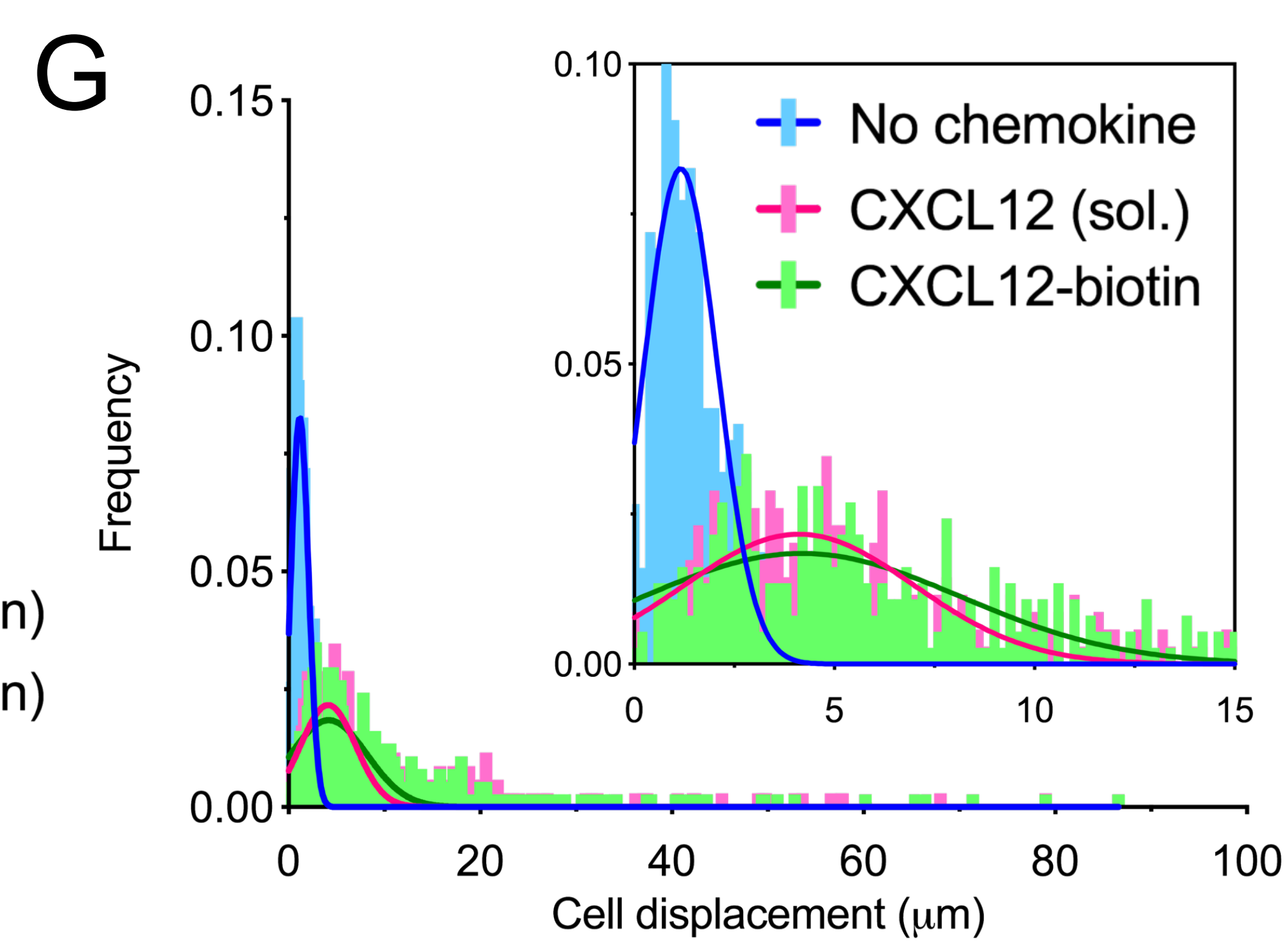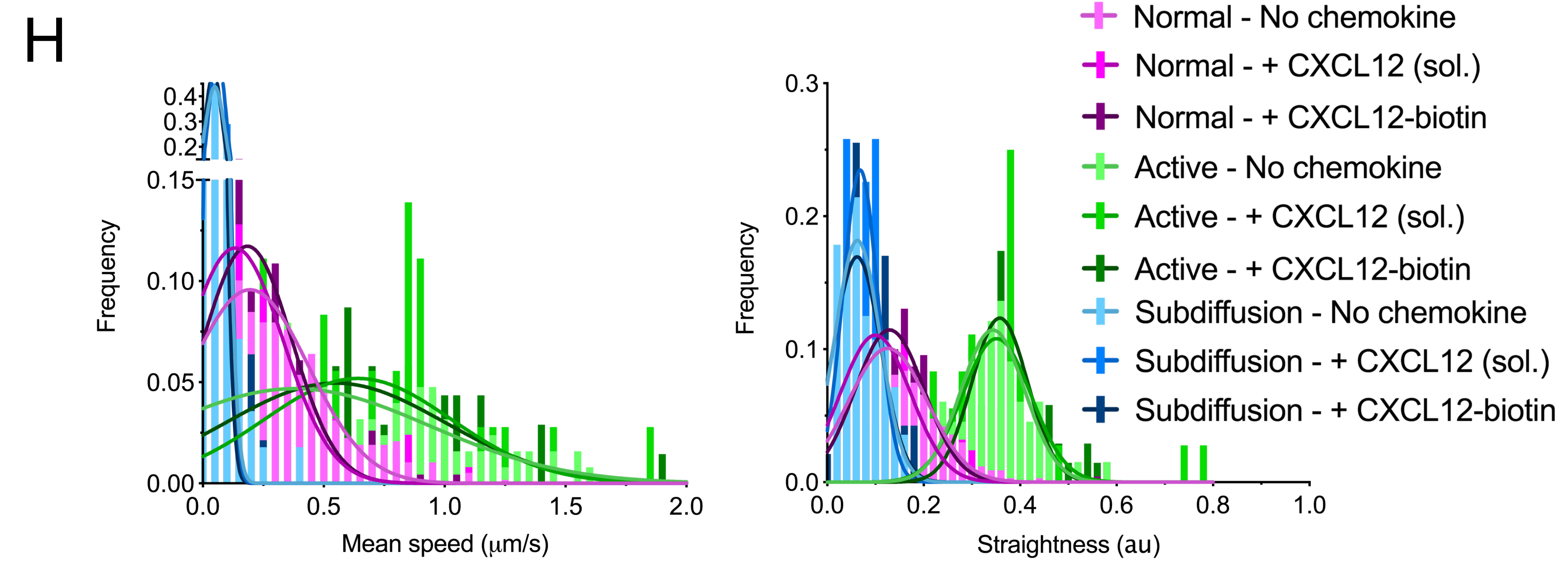

Supplement: Supplementary Figure 1 — (A) TIRFM image examples of primary CD4+ T cell blasts expressing CXCR4-HaloTag interacting with UCHT1- and ICAM1-containing SLBs for 5, 10, and 30 min. (B) Max-intensity projections and orthogonal views of a confocal microscopy z-stack of CD4+ T cell blast expressing CXCR4-HaloTag on SLB presenting ICAM1 and UCHT1 for 30 min. (C) TIRFM image examples of primary CD4+ T cell blasts prestained with anti-CXCR4 mAb interacting with UCHT1- and ICAM1-containing SLBs for 30 min. (D) TIRFM image examples of HA-DRB1-specific CD4+ T cells expressing CXCR4-HaloTag and pre-stained with anti-TCR mAb interacting with SLB presenting ICAM1 and pHA-MHC for 10 and 30 min. (E) TIRFM images (left), cross-sectional normalized intensity profile (center), and radial averages (right) for CD4+ T cell blasts expressing CXCR4-HaloTag and stained for F-actin with phalloidin, on activating SLB for 10 min and treated with indicated inhibitors. Plots are mean normalized intensity at each position ± std dev. (F) Histogram of straightness for single particle CXCR4-HaloTag trajectories with different diffusive properties under activating and non-activating conditions. (G) Histogram of cell displacement over 20 min incubation for CD4+ T cell blasts on ICAM1-containing SLB in the presence of soluble and surface-presented CXCL12, measured by time-lapse IRM. (H) Histograms of mean diffusion speed and straightness for single particle CXCR4-HaloTag trajectories with different diffusive properties under activating and conditions in the presence of soluble and surface-presented CXCL12. All scale bars are 5 μm. [file Image_1.PDF]

A

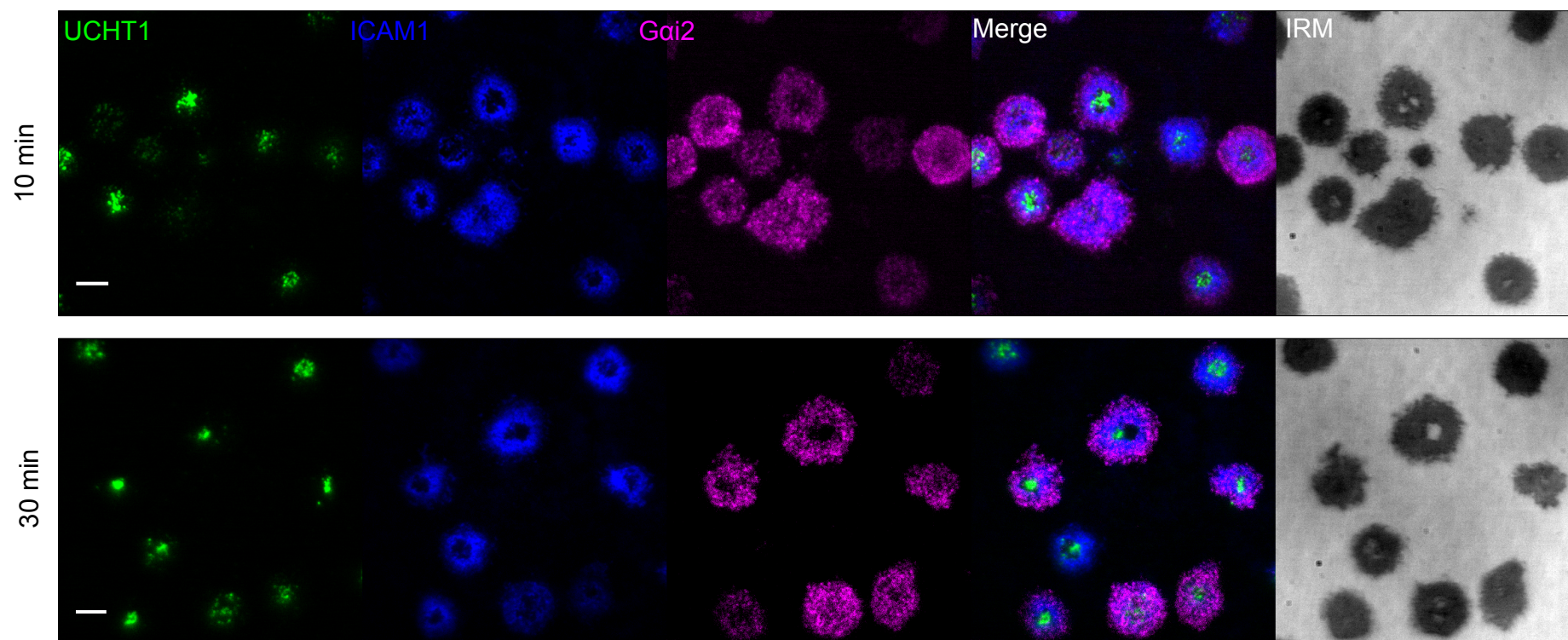

B

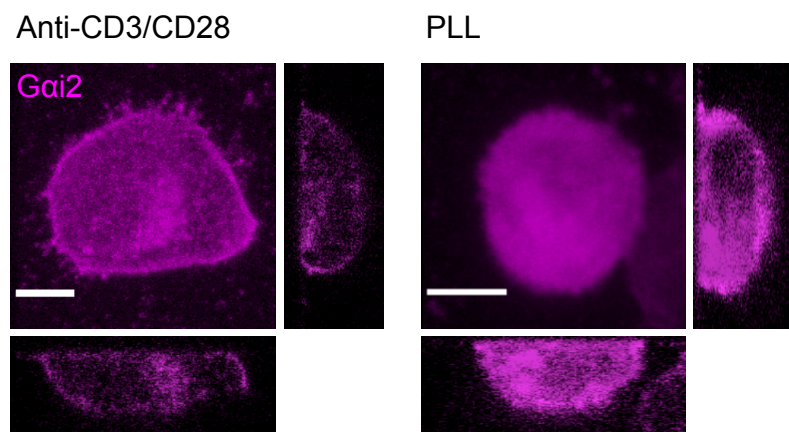

C

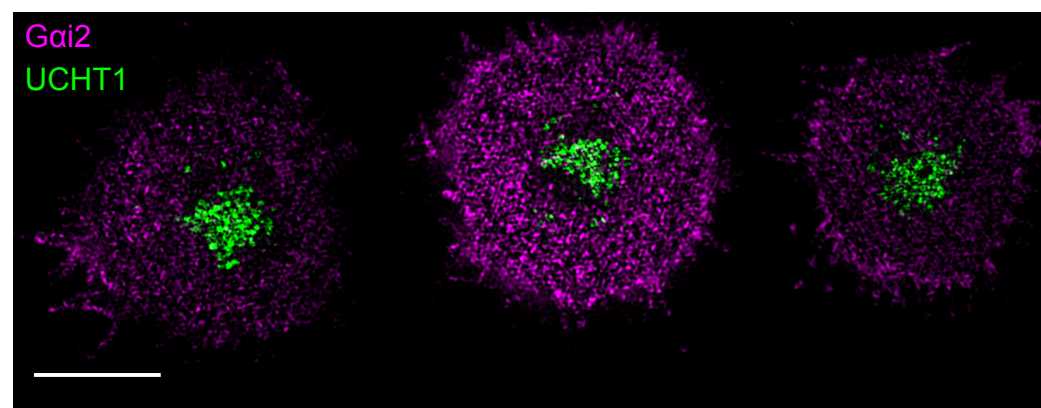

Supplement: Supplementary Figure 3 — (A) Representative TIRFM examples of Gαi2-SNAP-tag-expressing CD4+ T cell blasts interacting with or activating SLB for 10 or 30 min. (B) Max-intensity projections and orthogonal views of confocal microscopy z-stacks of CD4+ T cell blasts on glass coated with PLL or anti-CD3/CD28 antibodies. (C) TIRF-SIM examples of Gαi2-SNAP-tag in CD4+ T cell blasts on activating SLB for 10 min. All scale bars are 5 μm. [file Image_3.PDF]

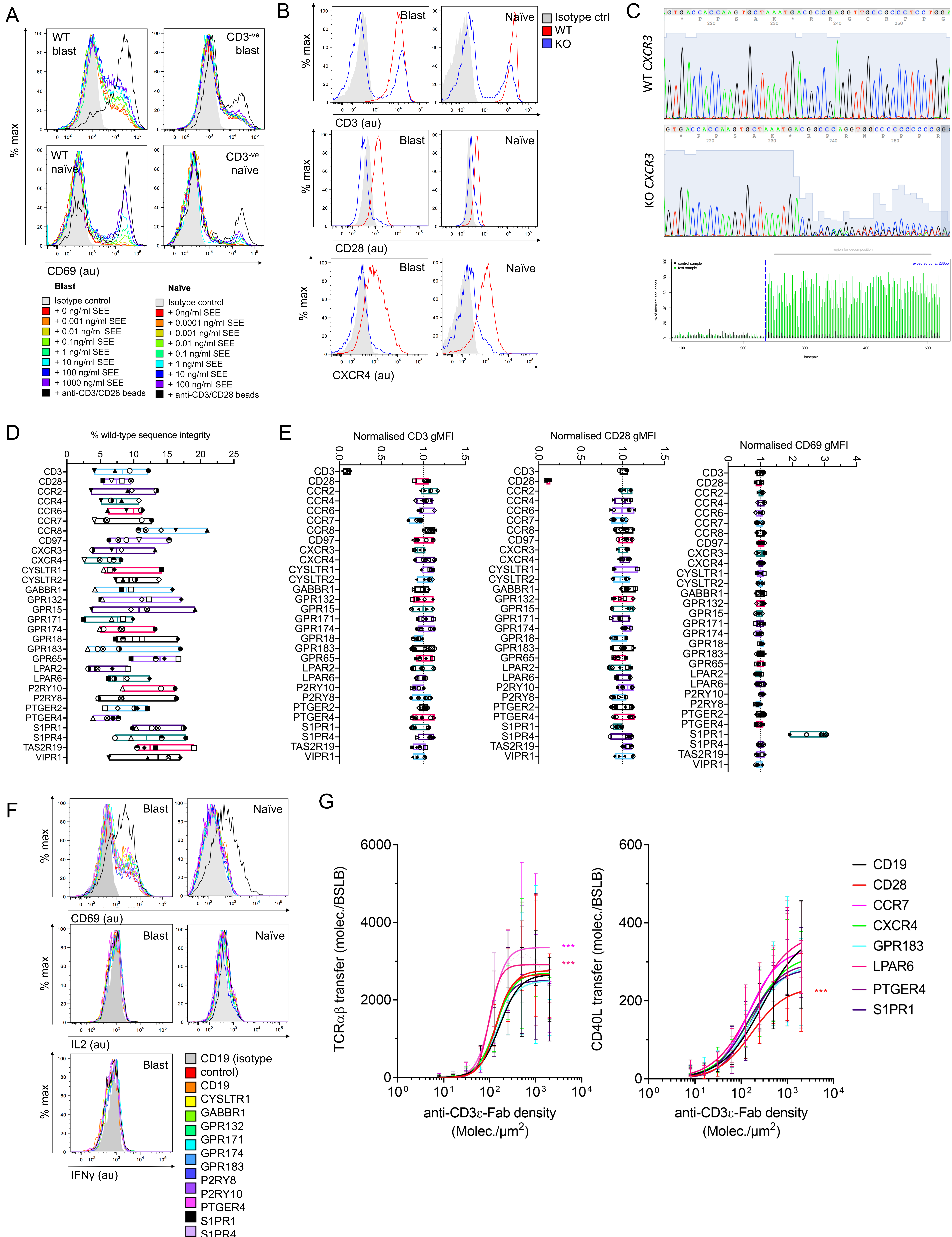

Supplement: Supplementary Figure 4 — (A) Example histograms of flow cytometry data of anti-CD69 staining on wt and CD3−ve CD4+ T cells (blast and naïve) from the same donor following 6 h stimulation with anti-CD3/CD28 beads or APCs loaded with the indicated concentration of SEE. (B) Example flow cytometry data for CD3, CD28, or CXCR4 expression in wt CD4+ T cells (blast and naïve) and cells transfected with Cas9 RNP complexes targeting the relevant gene (“KO”). (C) Example sequencing chromatograms used for TIDE analysis. CXCR3 sequences in wt and CXCR3 KO cells were compared to identify regions of aberrant signal (bottom), which then underwent decomposition to determine the relative frequencies of different indel mutations. (D) Pooled TIDE data from all Cas9 screen experiments. Values indicate the percentage of the sequencing data that corresponds to fully wild-type sequence. Boxes are mean with min-max; each symbol indicates a different donor. (E) Normalized CD3, CD28, and CD69 expression levels as measured by flow cytometry in unstimulated CD4+ T cell blasts following knockout of the indicated genes. Values are normalized to the gMFI value in wt cells. Boxes are mean with min-max; each symbol indicates a different donor. (F) Example flow cytometry histograms of anti-CD69, -IL2, and -IFNγ staining on unstimulated wt and individual-gene knockout CD4+ T cells (blast and naïve) from a single donor. (G) Absolute transfer of TCRαβ (left) or CD40L (right) from CD4+ blasts to BSLBs presenting ICAM1, CD40, and titrated densities of UCHT1. Plots show mean ± std dev., with best-fit non-linear response curves for each target. ***p < 0.001. [file Image_4.PDF]
