# Supplementary material for: Single-Molecule, Super-Resolution, and Functional Analysis of G Protein-Coupled Receptor Behavior Within the T Cell Immunological Synapse
Source: Front Cell Dev Biol. 2021 Jan 18;8:608484. doi: 10.3389/fcell.2020.608484 (PMC7848080; doi:10.3389/fcell.2020.608484)
Supplement: Supplementary Figure 2 — (A) Example flow cytometry histogram of anti-CXCR4 staining on wt and CXCR4−ve CD4+ T cell blasts transfected with HaloTag-fused CXCR4 mutants. (B) Example flow cytometry histogram of anti-CXCR4 staining on wt and CXCR4−ve CD4+ T cell blasts transfected with untagged CXCR4 mutants. (C) Example flow cytometry histograms of anti-CD69, -IL2, and -IFNγ staining on wt and CXCR4−ve CD4+ T cell blasts transfected with untagged CXCR4 mutants and stimulated with anti-CD3/CD28 beads for 6 h. [file Image_2.PDF]

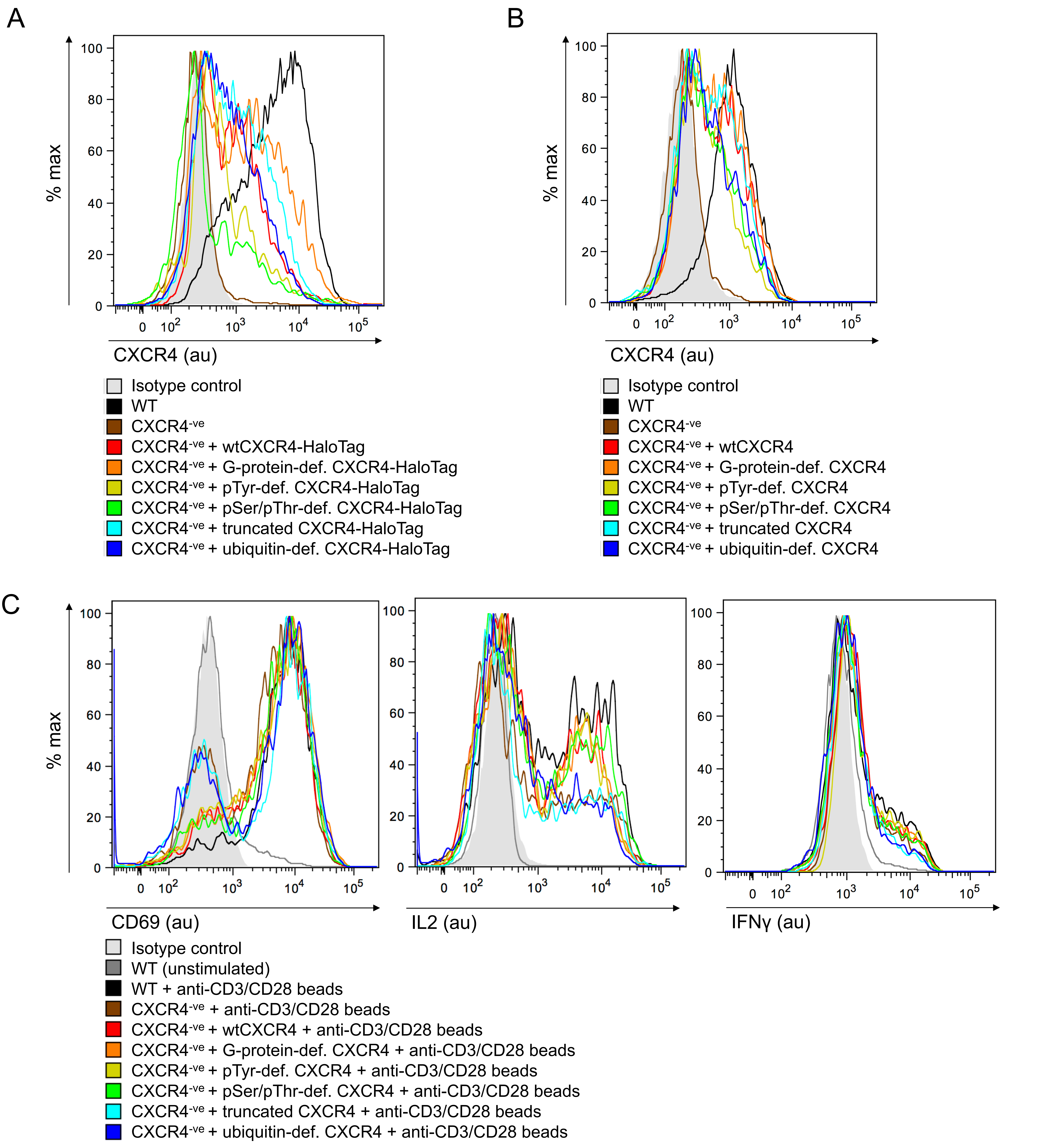
A
% max
CXCR4 (au)
Isotype control
WT
CXCR4^-ve
CXCR4^-ve + wtCXCR4-HaloTag
CXCR4^-ve + G-protein-def. CXCR4-HaloTag
CXCR4^-ve + pTyr-def. CXCR4-HaloTag
CXCR4^-ve + pSer/pThr-def. CXCR4-HaloTag
CXCR4^-ve + truncated CXCR4-HaloTag
CXCR4^-ve + ubiquitin-def. CXCR4-HaloTag
B
% max
CXCR4 (au)
Isotype control
WT
CXCR4^-ve
CXCR4^-ve + wtCXCR4
CXCR4^-ve + G-protein-def. CXCR4
CXCR4^-ve + pTyr-def. CXCR4
CXCR4^-ve + pSer/pThr-def. CXCR4
CXCR4^-ve + truncated CXCR4
CXCR4^-ve + ubiquitin-def. CXCR4
C
% max
CD69 (au)
IL2 (au)
IFNγ (au)
Isotype control
WT (unstimulated)
WT + anti-CD3/CD28 beads
CXCR4^-ve + anti-CD3/CD28 beads
CXCR4^-ve + wtCXCR4 + anti-CD3/CD28 beads
CXCR4^-ve + G-protein-def. CXCR4 + anti-CD3/CD28 beads
CXCR4^-ve + pTyr-def. CXCR4 + anti-CD3/CD28 beads
CXCR4^-ve + pSer/pThr-def. CXCR4 + anti-CD3/CD28 beads
CXCR4^-ve + truncated CXCR4 + anti-CD3/CD28 beads
CXCR4^-ve + ubiquitin-def. CXCR4 + anti-CD3/CD28 beads
